# PTEN‐induced kinase 1 is associated with renal aging, via the cGAS‐STING pathway

**DOI:** 10.1111/acel.13865

**Published:** 2023-05-15

**Authors:** Min Heui Ha, Man S. Kim, Hyun‐Ju An, Min‐Ji Sung, Yu Ho Lee, Dong‐Ho Yang, Sang Hyun Jung, Jihyun Baek, Yueun Choi, Deanne M. Taylor, Yuanchao Zhang, So‐Young Lee, Hye Yun Jeong

**Affiliations:** ^1^ Division of Nephrology, Department of Internal Medicine CHA Bundang Medical Center Seongnam Korea; ^2^ Clinical Research Institute Kyung Hee University Hospital at Gangdong, School of Medicine, Kyung Hee University Seoul Korea; ^3^ Department of Biomedical Science and Technology Graduate School, Kyung Hee University Seoul Korea; ^4^ Department of Pediatrics, Perelman School of Medicine University of Pennsylvania Philadelphia Pennsylvania USA; ^5^ Department of Biomedical and Health Informatics The Children's Hospital of Philadelphia Philadelphia Pennsylvania USA

**Keywords:** chronic kidney disease, mitochondria, PINK1, renal aging, STING

## Abstract

Mitochondrial dysfunction is considered to be an important mediator of the pro‐aging process in chronic kidney disease, which is continuously increasing worldwide. Although PTEN‐induced kinase 1 (PINK1) regulates mitochondrial function, its role in renal aging remains unclear. We investigated the association between PINK1 and renal aging, especially through the cGAS‐STING pathway, which is known to result in an inflammatory phenotype. Pink1 knockout (Pink1^−/−^) C57BL/6 mice and senescence‐induced renal tubular epithelial cells (HKC‐8) treated with H_2_O_2_ were used as the renal aging models. Extensive analyses at transcriptomic‐metabolic levels have explored changes in mitochondrial function in PINK1 deficiency. To investigate whether PINK1 deficiency affects renal aging through the cGAS‐STING pathway, we explored their expression levels in PINK1 knockout mice and senescence‐induced HKC‐8 cells. PINK1 deficiency enhances kidney fibrosis and tubular injury, and increases senescence and the senescence‐associated secretory phenotype (SASP). These phenomena were most apparent in the 24‐month‐old Pink1^−/−^ mice and HKC‐8 cells treated with PINK1 siRNA and H_2_O_2_. Gene expression analysis using RNA sequencing showed that PINK1 deficiency is associated with increased inflammatory responses, and transcriptomic and metabolomic analyses suggested that PINK1 deficiency is related to mitochondrial metabolic dysregulation. Activation of cGAS‐STING was prominent in the 24‐month‐old Pink1^−/−^ mice. The expression of SASPs was most noticeable in senescence‐induced HKC‐8 cells and was attenuated by the STING inhibitor, H151. PINK1 is associated with renal aging, and mitochondrial dysregulation by PINK1 deficiency might stimulate the cGAS‐STING pathway, eventually leading to senescence‐related inflammatory responses.

## Graphical abstract

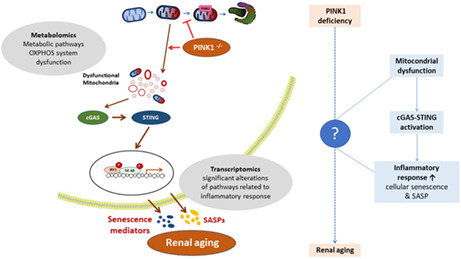

**Abbreviations**
ACR: albumin‐to‐creatinine ratio
CKD: chronic kidney disease
OCR: oxygen consumption rate
FN: fibronectin
PINK1: PTEN‐induced kinase 1
SASP: senescence‐associated secretory phenotype
TCA: tricarboxylic acid
TGF‐β1: transforming growth factor beta 1

## 1 INTRODUCTION

The increase in human life expectancy has led to a constant increase in the elderly population worldwide, which has led to medical, social, and economic problems, and investigations on aging and its prevention have continuously increased. Among the several aging‐related changes in the human organ system, progressive functional and structural deterioration of the kidney is known to be the most severe and is invariable across individuals (Weinstein & Anderson, 2010). With aging, the kidney experiences functional decline as well as histological changes, including interstitial fibrosis, tubular atrophy, and extracellular matrix accumulation. Aging involves cell senescence and the pathognomonic characteristics of the senescence‐associated secretory phenotype (SASP) (Fang et al., 2022). Although several studies have investigated the mechanism of age‐related dysfunctional changes in kidney function, the pathogenesis of renal aging remains unclear.

Recently, the role of mitophagy has been considered important in the pro‐aging process in chronic kidney disease (CKD) patients because the timely removal of dysfunctional mitochondria by mitophagy is crucial for kidney function regulation (Bhatia & Choi, 2019). Since dysfunctional mitochondria can induce inflammation, it can lead to chronic inflammation, which induces endothelial cell damage, cardiovascular events, and premature aging of the vascular wall in these patients (Carmona et al., 2020). PTEN‐induced kinase 1 (PINK1), a serine/threonine kinase, regulates mitochondrial dysfunction and initiates mitophagy (Durcan & Fon, 2015). However, the function of PINK1 in the aging process, especially in the kidney, remains unknown (Matsuda et al., 2013). A previous study showed that the *PINK1* gene deletion markedly increased the transforming growth factor beta 1 (TGF‐β1) expression in renal tubular cells, and renal fibrosis (Li et al., 2020). Another study also suggested that the loss of *PINK1* promoted profibrotic macrophagy (Bhatia et al., 2019), a critical contributor to renal inflammation and fibrosis (Bhatia & Choi, 2019). Among various inflammatory response‐related pathways, the cGAS‐STING pathway has emerged as a key mediator of inflammation, and a recent study on mouse heart tissue showed that PINK1 deficiency promotes mitochondrial DNA release and stimulates the cGAS‐STING pathway, resulting in an inflammatory phenotype (Sliter et al., 2018). However, few studies have investigated the correlation between PINK1 and the cGAS‐STING pathway in renal aging.

To examine the function of PINK‐1 in renal aging, we used *PINK1* knockout mice and senescence‐induced induced renal tubular epithelial (HKC‐8) cells, and showed that PINK1 deficiency significantly increased the hallmarks of senescence. Transcriptome level analysis showed that PINK1 deficiency enhanced inflammation‐associated pathways, and additional analysis at the metabolome level suggested that PINK1 deficiency was associated with mitochondrial dysregulation. Since the cGAS‐STING pathway is induced by mitochondrial dysfunction, and its activation is a crucial mediator of the inflammatory pathway, we additionally explored the change in the cGAS‐STING pathway, with respect to PINK1 expression.

## 2 RESULTS

### 2.1 PINK1 deficiency enhanced kidney fibrosis and tubular injury

**FIGURE 1:**
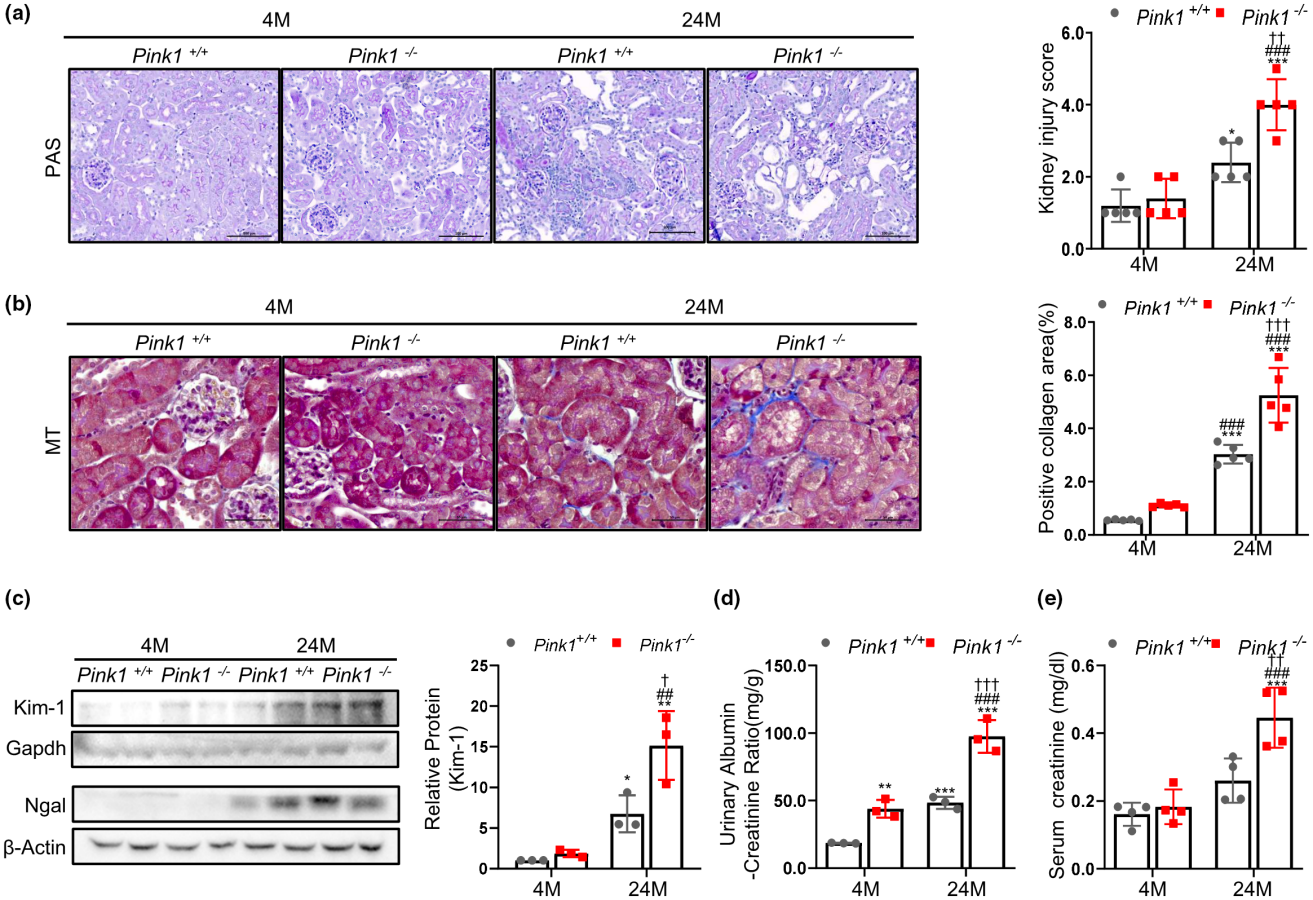
PINK1 deficiency increases renal fibrosis and tubular injury. (a) Representative photomicrographs of periodic acid‐Schiff‐stain of Pink1^+/+^ and Pink1^−/−^ mice kidneys at the age of 4 and 24 months. A semiquantitative assessment of renal tubular injury score was performed. Scale bar = 100 μm. (b) Representative photomicrographs of Masson's Trichrome‐stained Pink1^+/+^ and Pink1^−/−^ mice kidneys at the age of 4 and 24 months. A semiquantitative assessment of renal fibrosis was performed. Scale bar = 50 μm. (c) Western blotting of kidney injury molecule‐1 (Kim‐1) and neutrophil gelatinase‐associated lipocalin (NGAL). (d) Quantification of albuminuria in Pink1^+/+^ and Pink1^−/−^ mice at the age of 4 and 24 months. (e) Measurement of serum creatinine in Pink1^+/+^ and Pink1^−/−^ mice kidneys at the age of 4 and 24 months. Mean ± standard error of mean. *p < 0.05, **p < 0.01, ***p < 0.001 vs. Pink1^+/+^ 4 M, ^#^ *p* < 0.05, ^##^ *p* < 0.01, ^###^ *p* < 0.001 vs. Pink1^−/−^ 4 M, ^†^ *p* < 0.05, ^††^ *p* < 0.01, ^†††^ *p* < 0.001 vs. Pink1^+/+^ 24 M, M, months.

To determine the role of PINK1 in the aging process, we compared Pink1^−/−^ mice of age 4 and 24 months, to Pink1^+/+^ mice aged 4 and 24 months. Compared to the control group, renal tubular injury in PINK1^−/−^ mice with periodic acid‐Schiff staining was significantly more severe, and was more noticeable in 24‐month‐old Pink1^−/−^ mice (Figure 1a). In the Masson's Trichrome stained kidney tissues from Pink1^+/+^ and Pink1^−/−^ mice, the interstitial fibrotic regions of Pink1^−/−^ mice were characterized by markedly increased collagen deposition. Moreover, this feature was more noticeable in the 24‐month‐old Pink1^−/−^ mice (Figure 1b). Additionally, we observed an increase in the expressions of both the kidney injury molecule‐1 (Kim‐1) and NGAL, in the 24‐month‐old Pink1^−/−^ mice, as determined by the Western blot analysis (Figure 1c). Albuminuria, estimated by the urinary ACR and blood creatinine level, also increased in the 24‐month‐old mice, compared to the 4‐month‐old mice, and was most distinct in the 24‐month‐old Pink1^−/−^ mice (Figure 1d,e).

To clarify whether the effect of PINK1 knockout on the other cell types contributed to the change of aging, we also compared glomerulosclerosis and podocyte loss according to Pink1 knockout. The glomerular scoring for glomerulosclerosis significantly increased in the 24‐month‐old mice, but there was no significant change between in 24‐month Pink1^+/+^ and Pink1^−/−^ mice (Figure S1). These tendencies were also demonstrated on podocyte (Figure S2).

These results suggest that PINK1 deficiency might play a role in the pathological changes and damage in aged kidneys, especially affecting on renal tubular injury.

### 2.2 PINK1 deficiency increase senescence and senescence‐associated secretory phenotypes, aggravating renal aging

**FIGURE 2:**
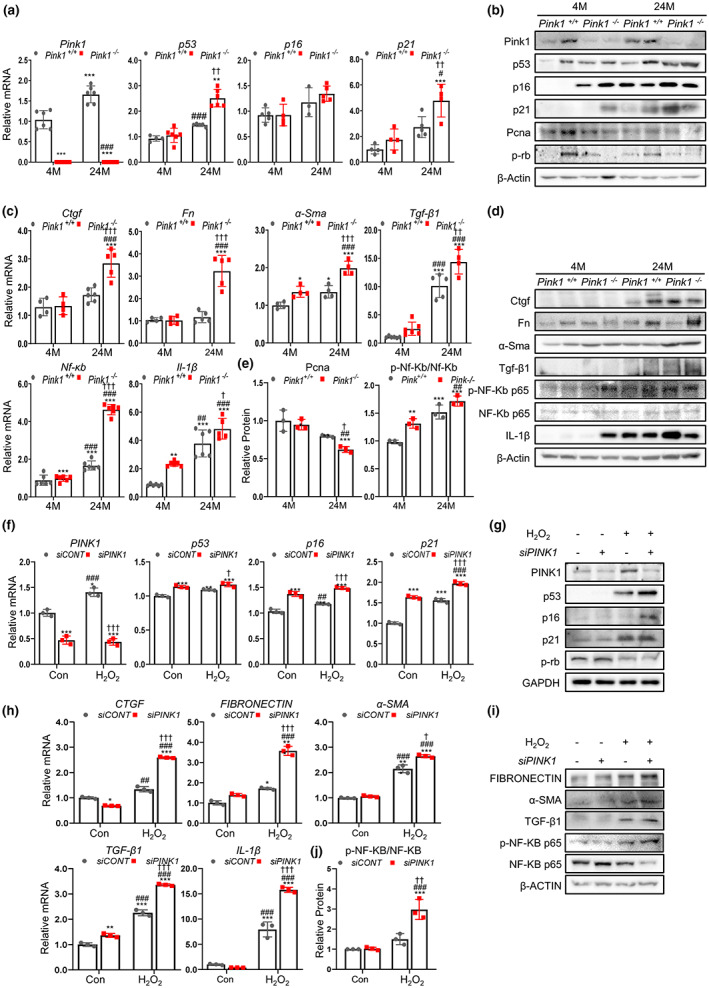
PINK1 deficiency increases senescence and senescence‐associated secretory phenotypes, aggravating renal aging and renal tubular epithelial cells. (a) The mRNA levels of senescence signaling mediators (p53, p16, and p21) in Pink1^+/+^ and Pink1^−/−^ mice at the age of 4 and 24 months. (b) Western blotting of senescence signaling mediators (p53, p16, and p21) and cell cycle markers (proliferating cell nuclear antigen (PCNA) and p‐Rb) in Pink1^+/+^ and Pink1^−/−^ mice at the age of 4 and 24 months. (c) The mRNA levels of SASPs (CTGF, Fn, a‐SMA, TGF‐β1, NF‐kb, and IL‐1β) in Pink1^+/+^ and Pink1^−/−^ mice at the age of 4 and 24 months. (d) Western blotting of SASPs (CTGF, Fn, a‐SMA, TGF‐β1, IL‐1β, NF‐kb, and p‐NF‐kb p65) in Pink1^+/+^ and Pink1^−/−^ mice at the age of 4 and 24 months. (e) Quantitative protein level of PCNA and p‐NF‐kb/NF‐kb for Western blot assay in Pink1^+/+^ and Pink1^−/−^ mice kidneys at the age of 4 and 24 months. (f) The mRNA levels of senescence signaling mediators, p53, p16, and p21, in siCONT and siPINK1 renal tubular epithelial cells (HKC‐8) with H_2_O_2_ treatment. (g) Western blotting of senescence signaling mediators (p53, p16, and p21) and a cell cycle marker (p‐Rb) in siCONT and siPINK1 H_2_O_2_‐treated HKC‐8 cells. (h) The mRNA level of SASPs (CTGF, Fibronectin, α‐SMA, TGF‐β1, and IL‐1β) in siCONT and siPINK1 H_2_O_2_‐treated HKC‐8 cells. (i) Western blotting of SASPs (Fibronectin, α‐SMA, TGF‐β1, p‐NF‐kb‐p65, and NF‐kb p65) in siCONT and siPINK1 H_2_O_2_‐treated HKC‐8 cells. (j) Quantitative protein level of p‐NF‐kb/NF‐kb for Western blot assay in siCONT and siPINK1 H_2_O_2_‐treated HKC‐8 cells. mean ± standard error of mean. (a, c, e) *p < 0.05, **p < 0.01, ***p < 0.001 vs. Pink1^+/+^ 4 M, ^#^ *p* < 0.05, ^##^ *p* < 0.01, ^###^ *p* < 0.001 vs. Pink1^−/−^ 4 M, ^†^ *p* < 0.05, ^††^ *p* < 0.01, ^†††^ *p* < 0.001 vs. Pink1^+/+^ 24 M, M, months. (f, h, j) *p < 0.05, **p < 0.01, ***p < 0.001 vs. siCONT without H_2_O_2_, ^#^ *p* < 0.05, ^##^ *p* < 0.01, ^###^ *p* < 0.001 vs. siPINK1 without H_2_O_2_, ^†^ *p* < 0.05, ^††^ *p* < 0.01, ^†††^ *p* < 0.001 vs. siCONT with H_2_O_2_, CONT, control.

To determine whether PINK1 deficiency is associated with renal aging, we also investigated changes in the senescence signaling mediator and SASP using Western blotting and qPCR. We observed that Pink1 expression levels were higher in 24‐month‐old Pink1^+/+^ mice, than in 4‐month‐old Pink1^+/+^ mice (Figure 2a). The senescence markers (p53, p16, p21, PCNA, and p‐Rb) significantly changed in the 24‐month‐old mice and were most prominent in the 24‐month‐old Pink1^−/−^ mice (Figure 2a,b,e). Similarly, the expression levels of pro‐inflammatory and pro‐fibrogenic SASP factors (CTGF, fibronectin, a‐SMA, TGF‐β1, NF‐kb, and IL‐1β) also increased in the 24‐month‐old mice and were more apparent in the 24‐month‐old Pink1^−/−^ mice (Figure 2c–e).

These results suggest that PINK1 might play a protective role in renal aging, and PINK1 deficiency could promote senescence signaling and SASP expression, which is involved in inflammatory and fibrogenic processes.

### 2.3 PINK1 knockdown promotes cellular senescence of renal tubular epithelial cells

To establish an in vitro senescence model, we used H_2_O_2_ treatment. To develop PINK1 knockdown cells, we transfected renal tubular epithelial cells with PINK1‐siRNA and explored the changes in senescence signaling mediators and SASP.

We found that the expression of these markers was significantly increased in the H_2_O_2_ treated cells, and that these increases were prominent in the H_2_O_2_ treated siPINK1 cells. Similar to the results in the mouse model, senescence markers (p53, p16, and p21) were significantly increased in the senescence‐induced siPINK1 cells (Figure 2f,g). The expression levels of pro‐inflammatory and pro‐fibrogenic SASP factors were also highest in the H_2_O_2_‐treated siPINK1 cells (Figure 2h,i,j).

Furthermore, we also investigated senescence signaling mediators and SASP in PINK1 overexpression to confirm the crucial role of the PINK1. The results showed that PINK1 overexpression significantly reversed the changes of these markers (Figure S3).

These data suggest that PINK1 in renal tubular epithelial cells is important for the regulation of cellular senescence.

### 2.4 Gene expression analysis using RNA‐seq highlights the relationship between PINK1 knockout and inflammatory responses

**FIGURE 3:**
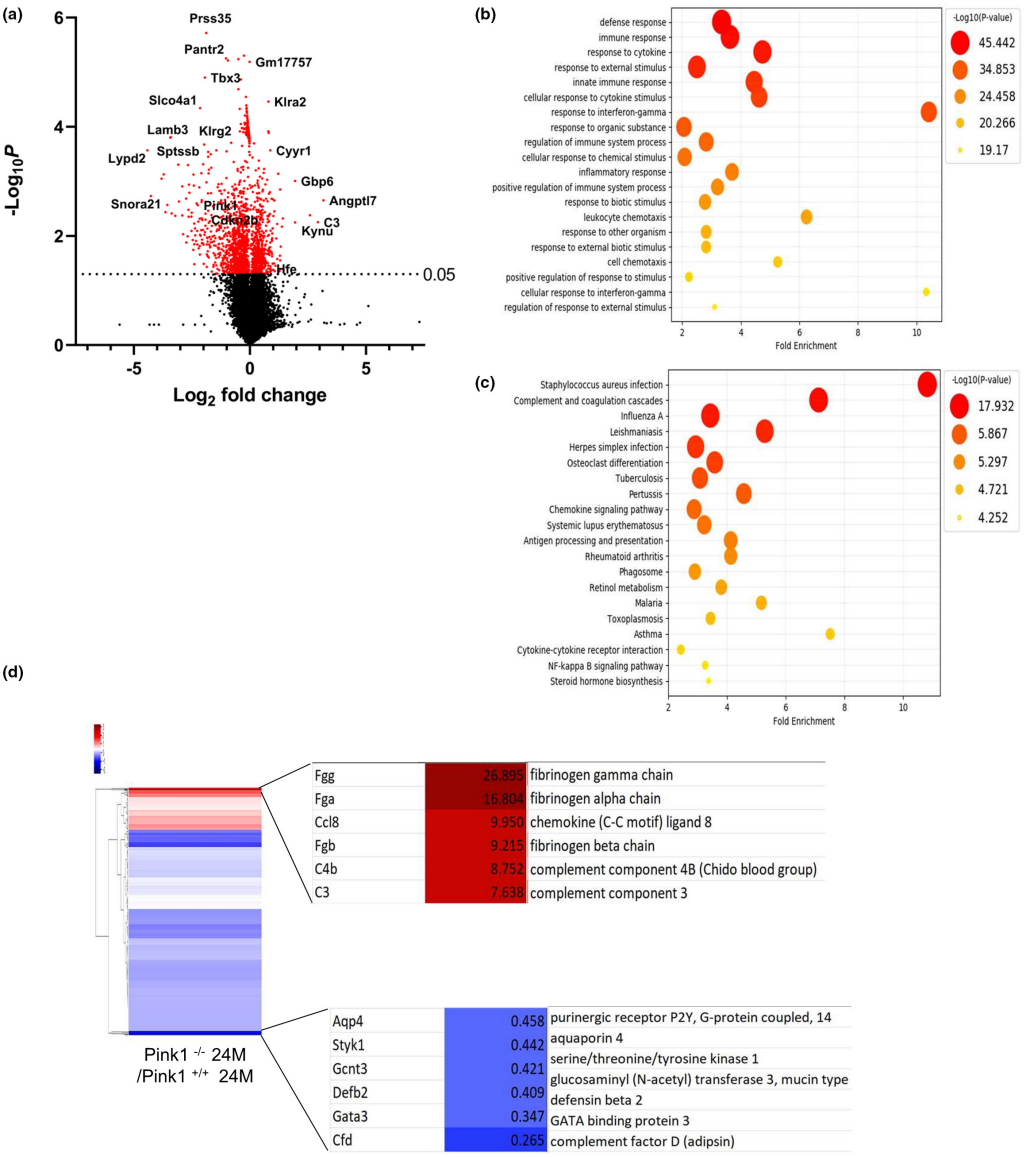
Gene enrichment analysis comparing Pink1^+/+^ and Pink1^−/−^ mice at 24 months. (a) The volcano plot shows statistically significant differentially expressed genes based on log2‐fold change of Pink1^−/−^ 24‐month‐old mice, compared to Pink1^+/+^ 24 month‐old mice. The significant differentially expressed proteins are shaded in red, and the significance threshold at *p*‐value 0.05 is indicated by the dashed line. (b) The GO analysis and (c) KEGG pathway enrichment analysis of significantly expressed proteins (*p* < 0.05). The bubble graphs of each presents top 20 most functionally enriched pathways. The data were analyzed using DAVID bioinformatics tools. (d) Heatmap of top 6 significantly increased (red) or decreased (blue) genes associated with inflammatory responses between Pink1^−/−^ 24‐month‐old mice and Pink1^+/+^ 24‐month‐old mice.

To gain insight into the mechanism through which PINK1 influences renal aging, we performed not only an extensive analysis at the transcriptomic level, but also a context‐specific genome‐scale simulation at the metabolic level, using RNA‐seq datasets (da Silveira et al., 2020) to investigate whether the loss of PINK1 could influence the aging process in the presence of a dysregulated mitochondria, which is known to be one of the triggering factors of the cGAS‐STING pathway (Smith, 2020). To reveal the underlying systemic impact of PINK1 deficiency in aging mice, we performed differential analyses on the sequencing data, including gene set enrichment analysis (GSEA). The volcano plot comparing the 24‐month‐old Pink1^+/+^ and Pink1^−/−^ mice demonstrated significantly, the presence of differentially expressed genes, indicated in red, whose cutoffs were ≥2 log2 fold change and *p* < 0.05 (Figure 3a). In addition, enrichment maps of the GO and KEGG sets were used for pathway enrichment analysis. From the GO analysis, it was estimated that pathways such as response to cytokines, cellular response to cytokine stimulus, regulation of immune system processes, inflammatory response, and cellular response to interferon‐gamma were significantly increased in the 24‐month‐old Pink1^−/−^ mice (Figure 3b). In the KEGG pathway analysis, we predicted the top 20 activated pathways, including complement cascades, chemokine signaling pathways, cytokine interactions, and the NF‐KB signaling pathway (Figure 3c). In Figure 3d, the heatmap shows the distribution of significant upregulated or downregulated differentially expressed genes associated with inflammatory responses, between the 24‐month‐old Pink1^+/+^ and Pink1^−/−^ mice.

### 2.5 Gene expression patterns suggest the relation between PINK1 knockout and mitochondrial dysfunction

**FIGURE 4:**
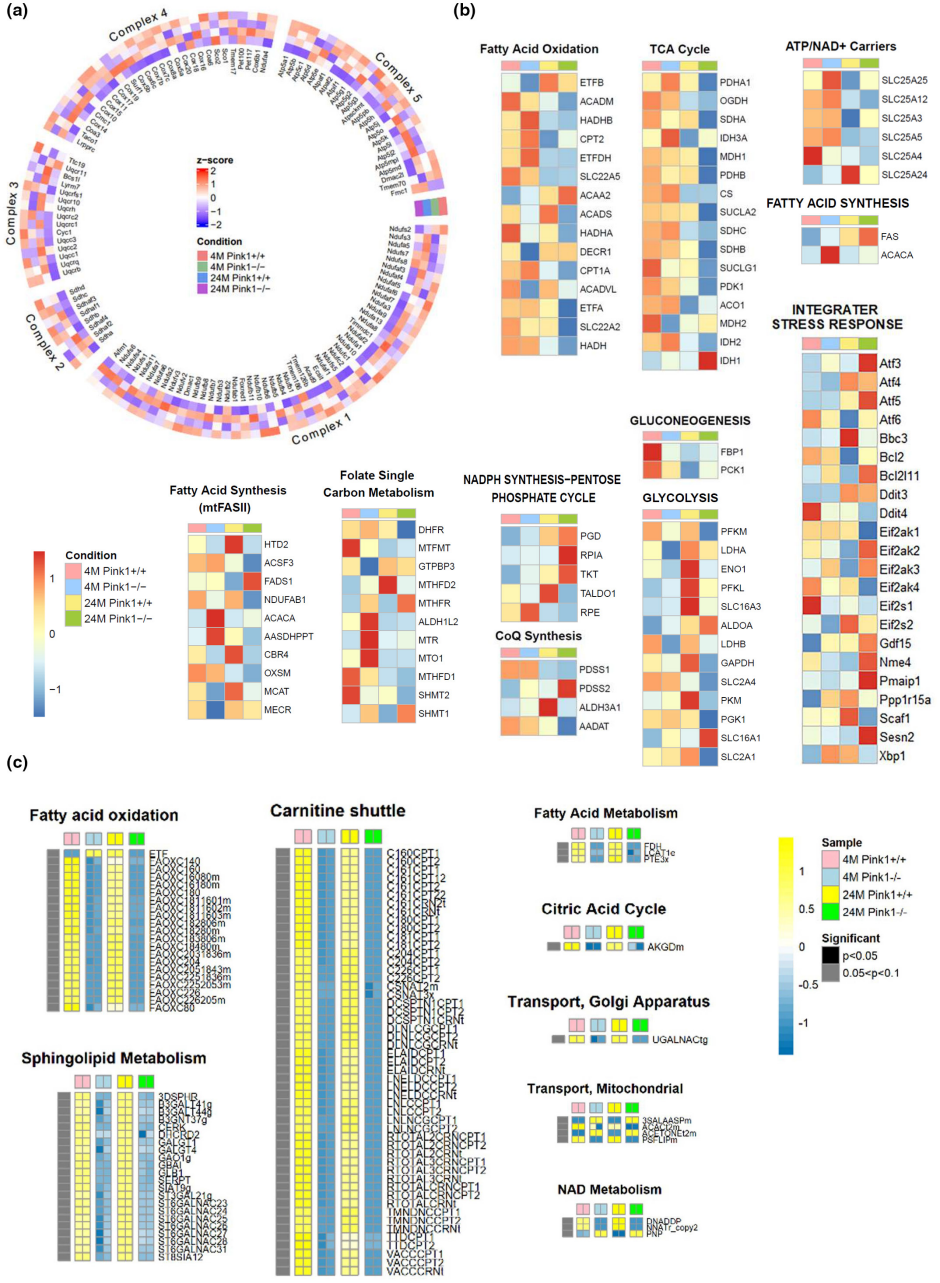
Transcriptional change and metabolic simulation comparing in Pink1^+/+^ and Pink1^−/−^ mice at 4 and 24 months. (a) The circular heatmap demonstrates the z‐score statistics of gene expression of mitochondrial complexes, comparing Pink1^−/−^ to Pink1^+/+^ mice, at the age of 4 and 24 months. Statistical significance was determined using the z‐score. (b) The differential expression of mitochondrial respiration and biogenetic pathways. Analysis on mitochondrial fatty acid oxidation, TCA cycle, ATP/ADP‐Pi exchange, fatty acid synthesis, mitochondrial fatty acid synthesis (mtFASII), folate metabolism, NADPH synthesis, coenzyme Q biosynthesis, gluconeogenesis, glycolysis and integrater stress response comparing Pink1^−/−^ to Pink1^+/+^ mice at the age of 4 and 24 months. Statistical significance was determined using the z‐score. (c) The heatmaps show flux values of mitochondrial metabolic pathways from Pink1^+/+^ and Pink1^−/−^ mice at the age of 4 and 24 months. The leftmost bar indicates fluxes whose results correspond to *p* < 0.05 (black) or *p* values between 0.05 and 0.1 (gray). Heatmap color scales indicate row‐wise Z‐scores for a particular flux.

To further examine the alterations in mitochondrial regulation in Pink1^−/−^ mice, we compared the transcriptional changes between Pink1^+/+^ and Pink1^−/−^ mice at 4 and 24 months. The Figure 4a shows a list of genes associated with mitochondrial complexes, and the expression of the genes related to OXPHOS complexes was significantly perturbed in the 24‐month‐old Pink1^−/−^ mice. Our transcriptomic analysis demonstrated that the assembly factors Ndufat7, Ndufb4, and Foxred1 were downregulated in complex I, whereas Tmem 186 in complex I, and Sco2 and Cox18 in complex IV were significantly upregulated in the 24‐month‐old Pink1^−/−^ mice.

We further focused on transcriptional changes in mitochondrial respiration and biogenetic pathways, the mitochondria‐associated pathways of which are illustrated in Figure 4b. SLC25A4, which is critical for mitochondrial energy production, were significantly downregulated in mitochondrial ATP/ADP‐Pi exchange in the 24‐month‐old Pink1^−/−^ mice. Pyruvate dehydrogenase E1 subunit alpha1, pyruvate dehydrogenase E1 subunit beta, and subunits of pyruvate dehydrogenase are the primary links between the tricarboxylic acid (TCA) cycle and glycolysis. Analysis of the TCA cycle revealed a significantly reduced expression of these subunits. The various genes associated with mitochondrial biogenesis, such as fatty acid synthesis, glycolysis, gluconeogenesis, and NADPH synthesis, were also significantly downregulated in the Pink1^−/−^ mice at 24 month. The analysis of integrater stress response showed that the genes involved in the response to stress such as ROS significantly increased in 24‐month‐old Pink1−/− mice (Figure 4b).

Additional analysis of mitochondrial localization and their regulatory activities by pathway was performed based on MitoCarta3.0 (Figure S4). We found that mitochondrial metabolism, dynamics, and protein import were significantly enriched with MitoCarta3.0 gene lists in the 24‐month‐old Pink1^−/−^ mice, where the enrichment score per metabolic pathway was computed through fGSEA.

To validate the results based on the transcriptomic‐metabolic level analyses, we further investigated the genes which was significantly changed on transcriptomic level analyses. The 9 genes in fatty acid oxidation, ATP/NAD+ carriers, CoQ synthesis, gluconeogenesis, mitochondrial fatty acid synthesis, and Glycolysis/gluconeogenesis were selected and these were validated through qPCR, consistent with omics data (Figure S5).

Hence, our analyses suggest that PINK1 deletion might affect the transcriptional inhibition of genes associated with mitochondrial OXPHOS, biogenesis, and dynamics, resulting in mitochondrial dysfunction.

### 2.6 Metabolic analysis suggests an association between PINK1 knockout and mitochondrial metabolic dysregulation

To explore metabolic variations necessary for mitochondria‐associated dysregulations, we ran a genome‐scale metabolic simulation comparing the four distinct mice groups. According to our metabolic simulation, as illustrated in Figure 4c, biological/enzymatic reactions in certain metabolic pathways demonstrated strong correlations with the gene expression patterns of mitochondria‐associated pathways at the transcriptome level. Flux comparisons between the control (Pink1^+/+^) and Pink1^−/−^ mice showed that some metabolic pathways, including fatty acid metabolism, like fatty acid oxidation, and sphingolipid metabolism, were significantly decreased in Pink1^−/−^ mice. The carnitine shuttle, citric acid cycle, mitochondrial transport, and NAD metabolism were also significantly suppressed in Pink1^−/−^ mice (Figure 4c). This metabolic simulation could provide additional insight into the metabolic dysregulation triggered by PINK1 deficiency.

### 2.7 PINK1 deficiency suppressed mitochondrial dynamics and mitophagy, increasing oxidative stress in mice renal aging

To investigate whether PINK1 deficiency induces renal aging in the context of mitochondrial dysfunction, we examined the changes in mitochondrial bioenergetic function and mitophagy in H_2_O_2_‐treated siPINK1 transfected cells, and compared the mitochondrial oxidative stress and dynamics in Pink1^+/+^ and Pink1^−/−^ mice at 4 and 24 months.

**FIGURE 5:**
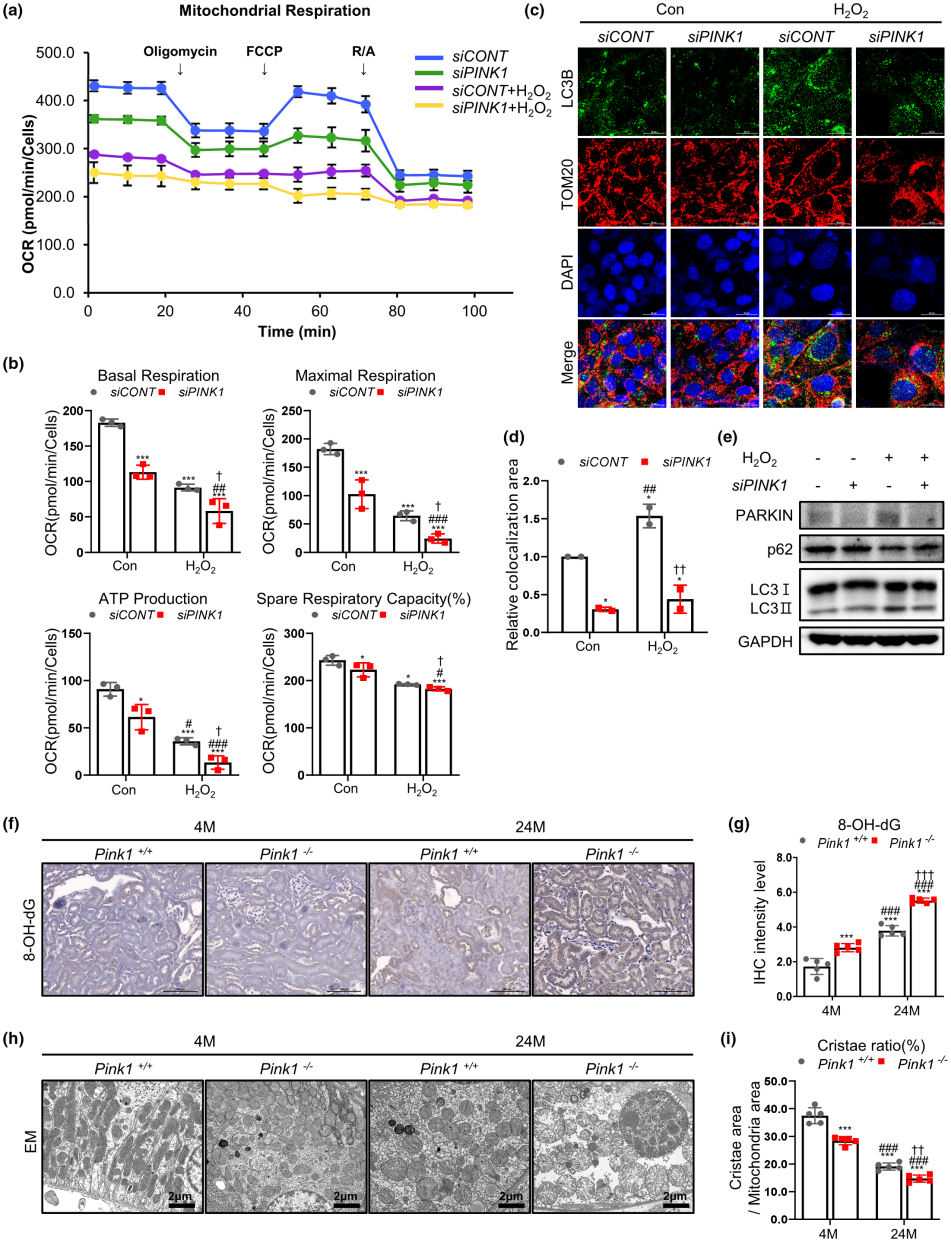
PINK1 deficiency leads to mitochondrial dysfunction in aging mice models and tubular epithelial cells treated with H_2_O_2_. (a) Measurement of seahorse XF cell mitochondrial oxygen consumption rate (OCR) test assay performed on siCONT and siPINK1 renal tubular epithelial cells treated with H_2_O_2_. (b) PINK1 deficiency affects basal respiration, maximal respiratory capacity, ATP production, and spare respiration capacity. (c, d) The comparison of LC3B and Tom20 colocalization in siPINK1 cells, to control cells, under H_2_O_2_ treatment. (e) Western blotting of mitophagy markers (Parkin, p62, LC3 I and II) in siCONT and siPINK1 H_2_O_2_‐treated HKC‐8 cells. (f, g) The levels of 8‐OH‐dG in Pink1^+/+^ and Pink1^−/−^ mice kidney at ages 4 and 24 months (h, i) Representative transmission electron microscopy images from Pink1^+/+^ and Pink1^−/−^ mice kidney at ages 4 and 24 months. Mean ± standard error of mean. (b, d) *p < 0.05, **p < 0.01, ***p < 0.001 vs. siCONT without H_2_O_2_, ^#^ *p* < 0.05, ^##^ *p* < 0.01, ^###^ *p* < 0.001 vs. siPINK1 without H_2_O_2_, ^†^ *p* < 0.05, ^††^ *p* < 0.01, ^†††^ *p* < 0.001 vs. siCONT with H_2_O_2_, CONT, control. (g, i) *p < 0.05, **p < 0.01, ***p < 0.001 vs. Pink1^+/+^ 4 M, ^#^ *p* < 0.05, ^##^ *p* < 0.01, ^###^ *p* < 0.001 vs. Pink1^−/−^ 4 M, ^†^ *p* < 0.05, ^††^ *p* < 0.01, ^†††^ *p* < 0.001 vs. Pink1^+/+^ 24 M, M, months.

For OCR measurement, the maximal respiration, ATP production, and respiratory capacity significantly declined in H_2_O_2_‐treated siRNA transfected cells. The reduced pattern of mitochondrial respiration was prominent in H_2_O_2_‐treated siPINK1 cells (Figure 5a,b). Additional analysis showed that H_2_O_2_ treatment induced LC3B and Tom20 co‐localization. However, this co‐localization decreased in H_2_O_2_‐treated siPINK1 cells, indicating reduced mitophagy in PINK1 deficiency (Figure 5c,d). In addition, decrease of LC3‐II accumulation was observed in H_2_O_2_‐treated siPINK1 cells (Figure 5e).

To further examine the effect of PINK1 deficiency on mitochondrial function in mouse renal aging, we analyzed mitochondrial oxidative stress under each condition. In animal model, the level of 8‐OH‐dG was significantly increased in Pink1^−/−^ mice, showing the most prominent change in 24‐month‐old Pink1^−/−^ mice (Figure 5f,g). The mitochondrial ROS measured by MitoSOX staining was also significantly amplified in H_2_O_2_‐treated siPINK1 (Figure S6).

Electron microscopy images showed that mitochondrial dynamics were significantly decreased in Pink1^−/−^ aging mice (Figure 5h,i). The mitochondria in 24‐month‐old Pink1^−/−^ mouse kidneys showed abnormally enlarged size and loss of cristae organization compared to control mice.

These data suggest that PINK1 deficiency might have an injurious effect on renal aging due to mitochondrial dysfunction, as reflected in our transcriptomic and metabolic analyses.

### 2.8 PINK1 deficiency promotes cGAS‐STING pathway activation in mice renal aging

**FIGURE 6:**
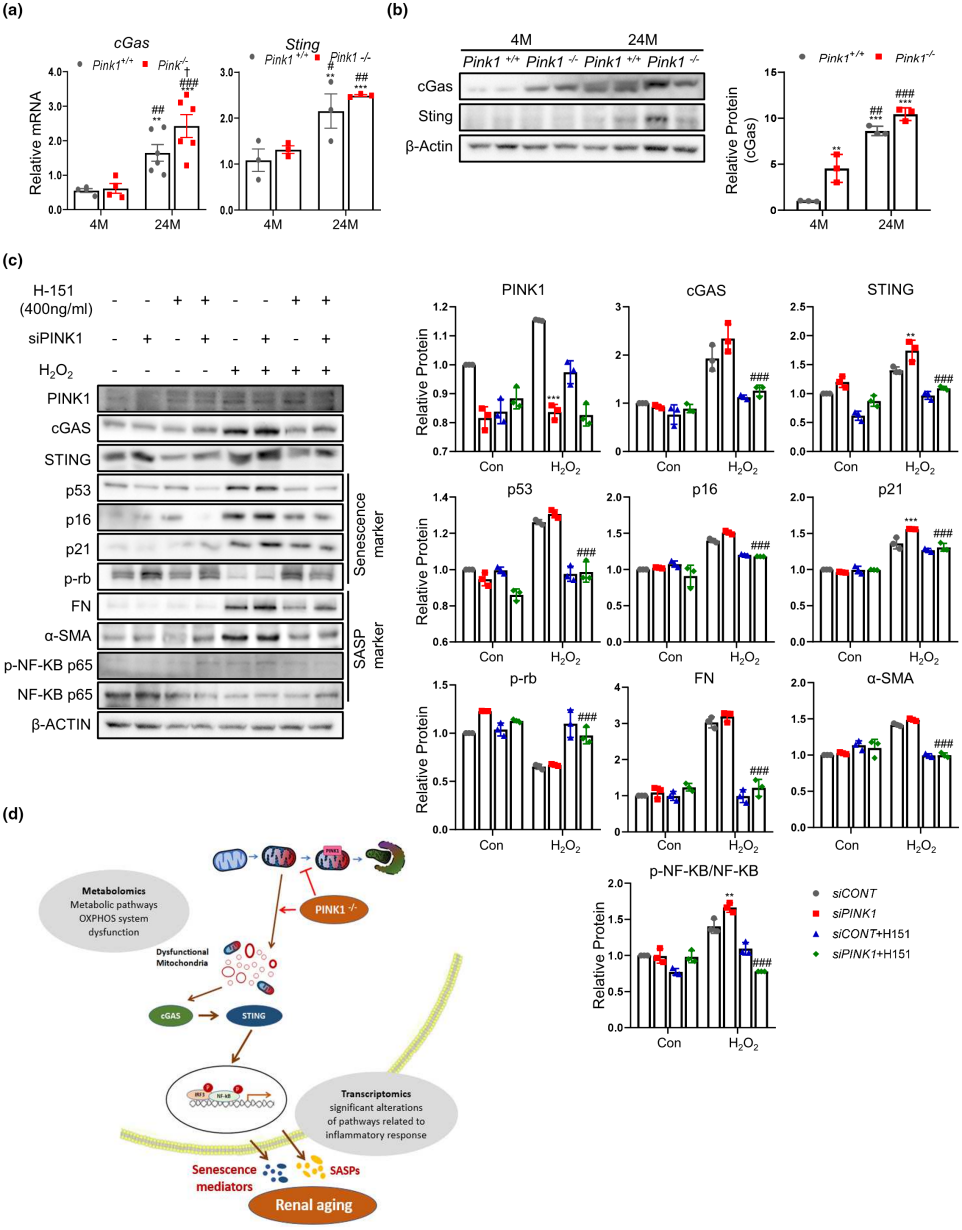
PINK1 deficiency activates cGAS‐STING pathway in aging mice model and tubular epithelial cells treated with H_2_O_2_. (a) The mRNA levels of cGAS and STING in Pink1^+/+^ and Pink1^−/−^ mice kidney tissue at 4 and 24 months. (b) Western blotting of cGAS and STING in Pink1^+/+^ and Pink1^−/−^ mice kidney tissue at 4 and 24 months. (c) The change of cGAS, STING, senescence signaling mediator and SASPs in siPINK1 and H_2_O_2_ treated cells on STING inhibitor, H‐151. (d) Schematic diagram summarizing role of PINK1 on renal aging. PINK1 deficiency enhances mitochondrial dysfunction known to be one of the STING activators, and eventually leads to renal aging presented by increased inflammatory response. Mean ± standard error of mean. (a) *p < 0.05, **p < 0.01, ***p < 0.001 vs. Pink1^+/+^ 4 M, ^#^ *p* < 0.05, ^##^ *p* < 0.01, ^###^ *p* < 0.001 vs. Pink1^−/−^ 4 M, ^†^ *p* < 0.05, ^††^ *p* < 0.01, ^†††^ *p* < 0.001 vs. Pink1^+/+^ 24 M, M, months (c) *p < 0.05, **p < 0.01, ***p < 0.001 vs. siCONT+H_2_O_2_, ^#^ *p* < 0.05, ^##^ *p* < 0.01, ^###^ *p* < 0.001 vs. siPINK1+ H_2_O_2_, CONT, control.

It has been suggested that mitochondrial dysfunction activates the cGAS‐STING pathway, which is one of the key mediators of the inflammatory response. To explore whether cGAS‐STING pathway activation is involved in renal aging and whether PINK1 deficiency affects the activation of this pathway, we compared the expression of cGAS and STING in Pink1^+/+^ and Pink1^−/−^ mouse kidney tissues at 4and 24 months. The cGAS‐STING signaling pathway was significantly activated in 24‐month‐old mice, compared to the 4‐month‐old mice, and PINK1 deficiency significantly promoted this activation (Figure 6a,b). These results suggest that the cGAS‐STING signaling pathway is involved in renal aging, and PINK1 deficiency may induce this signaling.

### 2.9 PINK1 deficiency activates cGAS‐STING pathway in senescence‐induced tubular epithelial cells

To investigate whether PINK1 deficiency affects aging through the STING pathway, we used tubular epithelial cells and compared the activity of the cGAS‐STING pathway with respect to PINK1 expression. Tubular epithelial cells treated with H_2_O_2_ had higher levels of cGAS and STING. These changes were more pronounced in siPINK1 and H_2_O_2_‐treated cells. The expression levels of senescence signaling mediators and SASP were also the most remarkable in siPINK1 and H_2_O_2_‐treated cells (Figure 6c).

Furthermore, we used H‐151, a specific inhibitor of STING, to explore the effect of PINK1 deficiency on cellular senescence. H‐151 treatment significantly reduced the expression of senescence‐signaling mediators and SASP (Figure 6c).

To exclude the non‐specificity of chemical inhibitor, we also used siSTING for genomic knockdown of STING. siSTING also significantly lowered the expression of senescence‐signaling mediators and SASP in siPINK1 and H_2_O_2_ treated cells (Figure S7).

As the results based on the transcriptomic analyses showed that the genes associated with OXPHOS complexes were significantly perturbed in the 24‐month‐old Pink1^−/−^ mice and the genes responsive to the stress such as ROS significantly increased in 24‐month‐old Pink1^−/−^ mice, we additionally explored whether this oxidative stress is associated with cGAS‐STING activation in PINK1 deficiency. The immunofluorescence staining showed noticeable co‐localization of 8‐OH‐dG and cGAS in 24‐month‐old Pink1^−/−^ mice, suggesting that oxidative stress induced by PINK1 deficiency might be involved in the activating cGAS (Figure S8).

These results suggest that PINK1 deficiency induces cellular senescence and is dependent on the cGAS‐STING pathway.

## 3 DISCUSSION

In this study, we showed that PINK1 deficiency was associated with enhanced renal aging, and the activation of the cGAS‐STING pathway was involved in this process. Extensive analyses of transcriptomic‐metabolic levels also demonstrated that mitochondrial dysregulation, known to activate STING, might be related to this mechanism through which PINK1 deficiency eventually induces renal aging (Figure 6d).

Cellular senescence is a histological hallmark of kidney aging (Hommos et al., 2017; O'Sullivan et al., 2017). The histological changes due to senescence, induced by SASP signaling, are known to be a fundamental factor promoting age‐related change in the kidney (Puelles et al., 2016; Wiggins et al., 2005). In this study, we have demonstrated that PINK1 deficiency aggravates age‐related changes in senescence signaling. In particular, the hallmark of kidney aging with increased SASP signaling was prominent in PINK1 deficiency (Fang et al., 2022).

PINK1 is crucial to mitochondrial quality control (Sato & Furuya, 2018). As PINK1‐dependent recruitment of parkin initiates mitophagy (Durcan & Fon, 2015; Vives‐Bauza et al., 2010), the loss of PINK1 could result in mitochondrial dysfunction. Although PNIK1 has been shown to be involved in the pathology of age‐related diseases like Parkinson disease or Alzheimer disease, the function of PINK1 in the aging process is not fully understood (Matsuda et al., 2013). Previous studies have suggested that aging induces PINK1 mediated mitophagy. Fiesel et al. (2015) showed that ubiquitin phosphorylation is promoted by PINK1 and accumulates in the elderly population and Pickrell et al. (2015) reported this phenomenon in mice. The loss of PINK1 caused shortening of lifespan in studies using Drosophila (Cornelissen et al., 2018; Pesah et al., 2004). However, studies on the direct function of PINK1 in aging, especially kidney aging, are rare. CKD is an important age‐related chronic disease characterized by the progressive loss of kidney function and renal fibrosis. The mechanism through which the uremic environment in CKD patients induces a pro‐aging process, involves mitochondrial dysfunction, decreased anti‐aging defenses, cellular senescence, and inflammation (Fang et al., 2022). Among these mechanisms, mitophagy has recently been considered to play a crucial role in mitochondrial quality control. The kidneys are rich in mitochondria, and dysfunctional mitochondria disrupt the cellular system causing cell death, and mitochondrial DNA from damaged cells recruits an inflammatory response, leading to further kidney damage (Bhatia et al., 2020).

Optimal mitophagy is important for the prevention of premature aging and early decline in kidney function. A study using an unilateral ureteral obstruction model demonstrated that loss of PINK1 accelerated renal extracellular matrix accumulation and kidney fibrosis (Bhatia et al., 2019). Another study using peripheral blood mononuclear stem cells from CKD patients reported lower PINK1 mRNA expression (Bhansali et al., 2017). A recent study also showed that loss of PINK1 aggravated kidney fibrosis, amplifying renal macrophages, which play integral roles in inflammation and subsequent kidney fibrosis progression, a pathognomonic manifestation of CKD (Bhatia et al., 2019). In conclusion, these results suggest that the PINK1‐associated pathway of mitophagy plays a pivotal role in regulating renal inflammation and fibrosis, providing evidence for the protective role of mitophagy in renal aging. In the present study, we also confirmed increased renal fibrosis and tubular injury in PINK1 knockout mice, and the more noticeable aging phenomenon was more prominent in PINK1 knockout mice.

Among the various mechanisms related to deleterious results induced by mitochondrial dysfunction, cGAS‐STING signaling has been suggested to be activated by DNA accumulation from damaged mitochondria, which promotes inflammatory cytokine production (Guo et al., 2021). Previous studies have demonstrated a senescence‐related role of the cGAS‐STING pathway. Guo et al. (2021) showed that the STING pathway promotes senescence and apoptosis in osteoarthritis. Wu et al. (2019) also suggested that the cGAS‐STING pathway is vital for early age‐related macular degeneration development. In addition, Yang et al. used cGAS deletion to abrogate senescence‐associated inflammatory gene expression and the senescence phenotype, suggesting a crucial role of cGAS in cellular senescence. In a previous study investigating the role of the cGAS‐STING pathway in renal disease, Maekawa et al. (2019) showed that mitochondrial dysfunction and subsequent activation of the cGAS‐STING pathway are important regulators of acute kidney injury. Another study also demonstrated that mitochondrial damage and STING pathway activation lead to renal inflammation and fibrosis, the pathognomonic phenomenon of chronic kidney disease (Chung et al., 2019).

Although studies focused on the direct correlation between PINK1 and the cGAS‐STING pathway are rare, a recent study on an in vivo mouse model found that mitochondrial stress led to a STING‐mediated inflammatory response in the absence of PINK1 (Sliter et al., 2018). They also demonstrated that the co‐deletion of PINK1 and STING could mitigate motor deficits and neuronal loss in a mouse model. In this study, we also investigated changes in the cGAS‐STING pathway in in vitro and in vivo models in the absence of PINK1 expression. Our results showed that PINK1 knockdown in aging cultured cells and a mouse model activated cGAS‐STING expression. In addition, STING deficiency alleviated the fibrotic and inflammatory changes induced by PINK1 knockout, suggesting significant involvement of the cGAS‐STING pathway in the renal aging process. Furthermore, the change in metabolic flux between normal aging and PINK1 knockout aging mice indicated that several key mitochondrial function‐related pathways changed according to PINK1 knockout status. Although we could not elucidate the direct correlation between PINK1 and the cGAS‐STING pathway under aging conditions, these results based on omics data analyses suggest that the effect of PINK1 knockout on aging might be related to mitochondrial dysregulation, which is known as the critical triggering factor of cGAS‐STING signaling (Li & Chen, 2018).

Our study reports transcriptomic‐metabolic level analyses comparing Pink1^+/+^ and Pink1^−/−^ mice. Our transcriptomic analysis showed the downregulation of various genes associated with mitochondrial respiration and biogenetic pathways, while the metabolic simulation‐based analysis showed significant alterations in the metabolism of PINK1 knockout mice, especially in metabolic pathways in which mitochondrial activity was directly associated.

Furthermore, the pathways involved in fatty acid metabolism were remarkably suppressed, and this result is similar to the patterns of current studies, which demonstrated that mitochondrial fatty acid oxidation dysfunction plays a role in the development of kidney fibrosis in CKD (Han et al., 2017; Kang et al., 2015; Simon & Hertig, 2015). Further studies investigating the underlying biological regulations through mass spectrometry‐based quantitative analysis might be helpful in identifying potential mitochondria‐associated metabolites with high selectivity and sensitivity.

In conclusion, PINK1 is associated with renal aging and stimulates the cGAS‐STING pathway, which might be mediated by dysregulated mitochondrial function inducing a cGAS‐STING‐related inflammatory response. Enhancing PINK1 activity might be a possible treatment target in renal aging, and further studies are needed to investigate PINK1 function in the cGAS‐STING signaling pathway, and as a promising therapeutic target for numerous aging‐related processes.

## 4 METHODS

### 4.1 Animal studies

*PINK1* knockout male C57BL/6 mice were obtained from the Center for Research Animals (Seoul, Korea) and maintained under a standard environment with a regular light/dark cycle and free access to water and a chow diet. In the intervention study, four groups of mice were used (*n* = 5 per group): (1) 4‐month‐old wild type (Pink1^+/+^); (2) 4‐month‐old *PINK1* knockout (Pink1^−/−^); (3) 24‐month‐old Pink1^+/+^; and (4) 24‐month‐old Pink1^−/−^. The mice were euthanized at ages 4 and 24 months, and their renal tissues were collected for analysis. All animal experiments were performed in accordance with the recommendations of the Guide for the Care and Use of Laboratory Animals of the Korea National Institutes of Health. All animal experiments were performed in accordance with the guidelines of the Animal Research Ethics Committee and the Institutional Animal Care and Use Committee of the CHA Bio Complex (No. 200012).

### 4.2 Cell culture

The human renal proximal tubular epithelial cell line, HKC‐8, was obtained from Dr. L. Rausen (Johns Hopkins University) and maintained in Dulbecco's modified eagle medium supplemented with Ham's F12 medium (DMEM/F12; WelGENE). The DMEM/F12 was supplemented with 5% fetal bovine serum (FBS) and 1% penicillin/streptomycin (WelGENE). To induce senescence, the HKC‐8 cells were treated with 200 μM hydrogen peroxide (H_2_O_2_) for 2 h, transferred to a fresh medium, and further incubated. After 48 h, the cells were collected for analysis.

### 4.3 Assessment of renal tissue morphology

Paraffin‐embedded mice kidney sections of 5 μm thickness were dewaxed in xylene and further rehydrated. For the histological assessment of the tubulointerstitial injury and fibrosis, the sections were stained with the periodic acid‐Schiff and Masson's trichrome reagents. Twenty high‐power fields of corticomedullary areas in each section were randomly selected for the analysis of the tubulointerstitial injury and fibrosis and were blindly assessed by an independent pathologist. The total tubular injury was graded on a scale of 0 to 4, based on the percentage of tubular dilatation and tubular epithelial cell destruction among the normal tubular cells (0: absent; 1: 1–25%; 2: 26–50%; 3: 50–75%; 4: >76%) in the affected tubular area. Fibrosis was expressed as a percentage of the positive area (20) in the total field, using computer‐assisted image analysis.

### 4.4 Analyses of the albumin‐to‐creatinine ratio (ACR)

To analyze the ACR in the urine, mouse urine was collected in a metabolic cage for 24 h. The collected urine samples were centrifuged (500 *g*, 5 min), and the supernatant was transferred to a clean microcentrifuge tube. The supernatant was then analyzed using an ACR assay kit (K551; BioVision Inc.), as per manufacturer's instructions.

### 4.5 Measurement of serum creatinine

Serum creatinine of Pink1^+/+^ and Pink1^−/−^ mice at ages 4 and 24 months was measured using a Creatinine Colorimetric/Fluorometric Assay Kit (K625‐100; BioVision), as per manufacturer's instructions. The absorbance was measured at 570 nm using a microplate reader.

### 4.6 Transfection of siRNA

Transient siRNA transfection was performed according to the protocol described by Lipofectamine® RNAiMAX Reagent. The PINK1 siRNA (sc‐44598; Santa Cruz Biotechnology) and STING siRNA (sc‐92042; Santa Cruz Biotechnology) were dissolved in siRNA buffer (20 mM KCl, 6 mM HEPES (pH 7.5), and 0.2 mM MgCl_2_), to prepare a 10 mM stock solution. Cells grown in six‐well plates were transfected with siRNAs, in a transfection medium (DMEM/F12 with 5% FBS and 1% penicillin–streptomycin) containing the liposomal transfection reagent Lipofectamine® RNAiMAX (Invitrogen). For each transfection, 200 μL of the transfection medium containing 10 mM siRNA stock solution was gently mixed with 200 μL of the transfection medium containing 10 μL of the transfection reagent. After incubating for 5 min at room temperature (24°C), siRNA‐lipid complexes were added to the cells in 1 mL transfection medium, and the cells were incubated for 24 h at 37°C. Cells at 40–60% confluence were transfected with siRNAs. After incubation in the siRNA (50 nM) solution for 24 h, the cells were treated with 200 μM hydrogen peroxide (H_2_O_2_) for 2 h. Then, the cells were transferred to a fresh medium and incubated for 48 h.

### 4.7 Construct of PINK1 overexpression cell line

To construct of stable PINK1 overexpression cell line, we transfected p‐Lenti‐PINK1‐mGFP‐P2A‐Puro (10 μg), 2nd generation lentiviral packaging plasmid (psPAX2, 10 μg) and VSV‐G envelope expressing plasmid (pMD.2G, 5 μg) use PEI to HEK293T. 2 days after, produced lentivirus was transduced to HKC‐8 use polybrene (8 μg/mL) for 24 h and then selected with 2 μg/mL puromycin.

### 4.8 Western blot analysis

The cells and kidney tissues were washed with phosphate‐buffered saline (PBS) and lysed in ice‐cold lysis buffer containing a protease inhibitor cocktail (Roche Diagnostics). The proteins were separated using SDS‐PAGE, with 8–12% SDS, and were transferred onto a polyvinylidene difluoride membrane (Millipore), through electroblotting. The membrane was blocked for 1 h at room temperature and was then incubated overnight at 4°C, with the primary antibodies: anti‐α‐SMA (ab5694, 1:1000; Abcam Inc.), anti‐fibronectin (ab2413, 1:1000; Abcam Inc.), anti‐TGF‐β1 (ab92486, 1:1000; Abcam Inc.), anti‐phospho‐NF‐κB p65 (#3033, 1:1000; Cell Signaling Technology), anti‐NF‐κB p65 (#8242, 1:1000; Cell Signaling Technology), anti‐KIM1 (#14971, 1:1000; Cell Signaling Technology), anti‐phospho‐Rb (#8516, 1:1000; Cell Signaling Technology), anti‐IL‐1β (sc‐52012, 1:1000; Santa Cruz Biotechnology, Inc.), anti‐STING (#13647, 1:1000; Cell Signaling Technology), anti‐cGAS (#15102, #31659 1:1000; Cell Signaling Technology), anti‐LC3B (#43566, 1:100; Cell Signaling Technology), anti‐SQSTM1/p62 (#5114, 1:1000; Cell signaling Technology), anti‐p53 (sc‐99, 1:1000; Santa Cruz Biotechnology, Inc.), anti‐p16 (sc‐1661, 1:1000; Santa Cruz Biotechnology, Inc.), anti‐p21 (sc‐6246, 1:1000; Santa Cruz Biotechnology, Inc.), anti‐CTGF (sc‐365790, 1:1000; Santa Cruz Biotechnology), anti‐PCNA (sc‐25280, 1:1000; Santa Cruz Biotechnology, Inc.), anti‐ neutrophil gelatinase‐associated lipocalin (NGAL) (sc‐515876, 1:1000; Santa Cruz Biotechnology, Inc.), anti‐PINK1 (sc‐517353, 1:1000; Santa Cruz Biotechnology, Inc.), and anti‐Parkin (sc‐32282, 1:1000; Santa Cruz Biotechnology, Inc.). Subsequently, the membranes were stained with horseradish peroxidase‐conjugated goat anti‐rabbit or anti‐mouse immunoglobulin G (ADI‐SAB‐300‐J and ADI‐SAB‐100‐J, respectively, 1:5000; Enzo Life Science, Inc.). Immunoreactive bands were detected using enhanced chemiluminescence (1705061, Bio‐Rad Laboratories, Inc.). Anti‐GAPDH (sc‐365062, 1:2000) and anti‐β‐actin (sc‐47778, 1:2000) antibodies (Santa Cruz Biotechnology, Inc.) were used as internal controls.

### 4.9 Isolation of total RNA and real‐time PCR (qPCR)

Total RNA was extracted from the kidney tissue or cultured kidney cells using the Total RNA Isolation Kit (Macherey‐Nagel), according to the manufacturer's instructions. The SensiFAST cDNA Synthesis Kit (Meridian Bioscience Inc.) was used for cDNA synthesis. Real‐time PCR was performed using the AMPIGENE® qPCR Green Mix Lo‐ROX (Enzo Life Sciences, Inc.). The primer sequences used for mRNA analysis are listed in the Table S1.

### 4.10 RNA‐seq preprocessing and transcriptomic analysis

We obtained the gene expression data for two independent libraries for Pink1^−/−^ and Pink1^+/+^ mice, at two different time points, 4 and 24 months, respectively, through a typical bulk RNA‐seq process, producing eight samples in total. Before mapping the RNA‐seq reads, quality control, including trimming, was performed using bbduk (BBMap v36.59) (Brian, n.d.) and FASTQC (v0.11.7) (Simon, 2010). We performed mapping‐quantification through a STAR‐HTSeq workflow, using STAR (v2.7.3a) (Dobin et al., 2013) and HTSeq‐count (v0.12.4) (Anders et al., 2015), where all sequencing reads of the four different conditions were aligned to the reference genome, GRCm39, along with its annotation. After obtaining the read counts of gene expression, we performed normalization and basic differential analysis on the levels utilizing the DESeq2 package (Love et al., 2014), with the embedded normalization method, variance stabilizing transformation (VST). Subsequently, we performed basic differential analyses using volcano plots, gene ontology (GO), and Kyoto Encyclopedia of Genes and Genomes (KEGG) ontology, by applying R packages such as EnhancedVolcano (Kevin et al., 2018) and clusterProfiler (Wu et al., 2021). The full dataset is available in the National Center for Biotechnology Information Gene Expression Omnibus database (NCBI GEO data base) with the accession number GSE 217775. Raw data are accessible at https://www.ncbi.nlm.nih.gov/geo/query/acc.cgi?acc=GSE217775.

### 4.11 Analysis on mitochondrial regulation

A custom‐made gene list on mitochondrial regulation (Guarnieri et al., 2022) was applied to selectively examine the gene expression patterns associated with mitochondrial activities, across mitochondria‐associated pathways. The list contains not only gene lists of mitochondrial complexes, as shown in Figure 4a, but also lists additional mitochondria‐associated pathways illustrated in Figure 4b. In addition, another list based on mitochondrial localization and their regulatory activities by pathways collected from MitoCarta3.0 (Rath et al., 2021), were assessed using pathway enrichment analysis, as described in Figure S4. In this analysis, the fast gene set enrichment analysis (fGSEA) (Korotkevich et al., 2021) package with its nominal enrichment score (NES) was applied.

### 4.12 Metabolic modeling

We computed the optimized flux levels of all available enzymatic reactions through the imm1415 metabolic model (Sigurdsson et al., 2010) for each RNA‐seq sample, using our modified metabolic simulation model, which was adopted from previously adjusted versions (da Silveira et al., 2020; Guarnieri et al., 2022) of the original model (Zhang et al., 2020). The simulation model is a context‐specific genome‐scale metabolic model that subsets all associations between genes and metabolic reactions from the imm1415 model (Sigurdsson et al., 2010), using cobrapy (Ebrahim et al., 2013) and CORDA (Schultz & Qutub, 2016). CORDA assigned confidence scores to reactions included in the model; the highest medium score, and lowest scores were 3, 2, and 1, respectively, which were applied to the top 35%, 35–90%, and 90–100% of the reactions, respectively. The confidence score distribution necessitated minimization of the standard deviation of the estimated fluxes across samples from each group, for all groups. Note that full activation on some vital pathways due to prerequisite for the simulation stability: “Oxidative Phosphorylation,” “Citric Acid Cycle,” “Glycolysis/Gluconeogenesis,” “CoA Biosynthesis,” “CoA Catabolism,” “NAD Metabolism,” “Fatty Acid Metabolism,” “Fatty acid activation,” “Fatty acid elongation,” “Fatty acid oxidation,” “ROS Detoxification,” and “Biomass and maintenance functions.” While the gene expression levels were mapped into the model for each sample, other parameters were constant for all simulations. Each simulation reported the flux levels of all activated reactions through flux balance analysis (FBA), optimizing each reaction as the objective, and the outcome as estimated flux levels were analyzed as grouped variables for the comparison between the conditions: Pink1^−/−^ at 4 months, Pink1^−/−^ at 24 months, Pink1^+/+^ at 4 months, and Pink1^+/+^ at 24 months. Since it is impossible to presume variance or normality between each condition, the non‐parametric Van der Waerden (VdW) test was applied to adequately compare their flux levels, using the R matrixTests package (v. 0.1.9). The comparison is presented as heatmaps with row‐wise Z‐scores on flux levels for each reaction in Figure 4c, where some pathways were chosen based on mitochondria‐associated connection or PINK1‐associated peculiarity, while their selected reactions with *p* < 0.05 were considered significant. The simulation process had an environment in which Jupyter notebook 6.1.5 on Python 3.7.7 (Kluyver, 2016), was utilized. Our metabolic model was optimized for ATP synthesis, to linear programming (LP), using Gurobi solver (https://www.gurobi.com) (“Gurobi Optimization, L.L.C.”, 2020). ATP synthesis capacity through all pathways in mice, was computed by estimating the optimized “ATPM” reaction flux level where “ATPM” is the pseudoreaction involved in ATP maintenance requirement.

### 4.13 Measurement of oxygen consumption rate (OCR)

The day before the assay, a sensor cartridge in Seahorse XF Calibrant was hydrated at 37°C in a non‐CO_2_ incubator, overnight. Senescence‐induced HKC‐8 cells were washed twice with pre‐warmed serum‐free XF DMEM medium (3151 mg/L D‐glucose, 365 mg/L L‐glutamine, 55 mg/L sodium pyruvate), pH 7.4, with a pipette, and each well was filled with 500 μL of serum‐free XF DMEM medium (Agilent). Seahorse XF Cell Mito Stress Kit (1.5 μM Oligomycin; 0.5 μM FCCP; and 0.5 μM Rotenone and antimycin) (Agilent) was placed in each port, and further placed at 37°C in a non‐CO_2_ incubator for 45 min, prior to the assay. OCR was measured using the Seahorse Cell Mito Stress Test with a Seahorse XFe24 Flux analyzer (Agilent). The data were analyzed using the Seahorse XFe Wave Software (Agilent).

### 4.14 Immunocytochemistry/immunofluorescence

For immunocytochemistry, HKC‐8 cells were fixed in 3.7% formaldehyde in PBS. Blocking was performed in 10% goat serum in PBS + 0.1% Tween20. The cells were labeled with the LC3B (#43566, 1:100; Cell Signaling Technology), and Tom20 (sc‐17764, 1:100; Santa Cruz Biotechnology, Inc.) antibodies. Immunofluorescence analysis was perform on paraffin‐embedded kidney section. The anti‐p21 (sc‐6246, 1:1000; Santa Cruz Biotechnology, Inc.), anti‐8‐hydroxydeoxyguanosine (8‐OH‐dG) (sc‐66036; 1:500; Santa Cruz Biotechnology, Inc.) anti‐Nephrin (ab136894, 1:100; Abcam Inc.), and anti‐cGAS (#31659, 1:100; Cell Signaling Technology) were used. Following antibody labeling; the cells were stained with Alexa Flour 488 (A‐11008, A‐32766, 1:1000; Invitrogen) and 594 (A‐11005, A‐32754 1:1000; Invitrogen). The HKC‐8 cells and kidney tissues were then counterstained with DAPI and examined using confocal microscopy (LSM‐700; Carl Zeiss).

### 4.15 Immunohistochemistry

Immunohistochemical analysis was performed on paraffin‐embedded kidneys sections of 5 μm thickness, according to the Polink‐2 HRP Broad with DAB Kit (D22‐18; GBI Labs) protocols. The primary mouse anti‐8‐hydroxydeoxyguanosine (anti‐8‐OH‐dG) antibody (sc‐66036; 1:500; Santa Cruz Biotechnology, Inc.) was used. Counterstaining was performed using hematoxylin.

### 4.16 Electron microscopy

Renal cortices of Pink1 ^+/+^ and Pink1^−/−^ mice aged 4 and 24 months were minced into 1 mm^3^ pieces and fixed overnight in 2% glutaraldehyde‐2% paraformaldehyde in 0.1 M phosphate buffer (pH 7.4). The minced pieces were imaged using the HT7800 transmission electron microscope (Hitachi High‐Tech).

### 4.17 Measurement of mitochondrial reactive oxygen species

To measure mitochondrial ROS, we used H_2_O_2_‐induced senescent HKC‐8 on siCONT and siPINK1. For analysis of MitoSOX (5 μM; Invitrogen), incubate cells for 30 min at 37°C and 5% CO_2_ were used. MitoSox red were detected with CytoFlex Flow cytometry (Beckman Coulter Life Sciences).

### 4.18 Statistics

The data were expressed as mean ± standard error of mean. Comparisons between groups were made using one‐way ANOVA, followed by the Student–Newman–Keuls test. Statistical significance was set at *p* < 0.05. All analyses were performed using the GraphPad Prism software (v8; GraphPad Software, Inc.).

## AUTHOR CONTRIBUTIONS

MHH: Conceptualization, data curation, formal analysis, and methodology. MSK: Data curation, formal analysis, original draft, and methodology. SYL: Conceptualization, data curation, formal analysis, and validation. HYJ: Original draft, formal analysis, funding acquisition, and validation. YHL: formal analysis and investigation. DHY: formal analysis and investigation. MJS: formal analysis and investigation. HJA: Formal analysis, investigation, and validation. SHJ: formal analysis and investigation. JHB: formal analysis and investigation. YEC: visualization. DT: visualization. YZ: visualization.

## CONFLICT OF INTEREST STATEMENT

The authors declare no conflicts of interest.

## Supporting information


Data S1:

Figure S1:

Figure S2:

Figure S3:

Figure S4:

Figure S5:

Figure S6:

Figure S7:

Figure S8:

## Data Availability

All data used in this study are available in this article.
