# Supplementary material for: PTEN‐induced kinase 1 is associated with renal aging, via the cGAS‐STING pathway
Source: Aging Cell. 2023 May 15;22(7):e13865. doi: 10.1111/acel.13865 (PMC10352563; doi:10.1111/acel.13865)
Supplement: Supplementary file 1 — Data S1: [file ACEL-22-e13865-s003.docx]

**Supplemental material**

Supplemental Table 1. Primer sequences for mRNA analyses

| Primer | | Primer sequence 5’→3’ | | | Amplification size |  |
| --- | --- | --- | --- | --- | --- | --- |
| *18s* | | Mouse | | Forward: CTGAGAAACGGCTACCACATC | 107 | |
|  |  |  |  | Reverse: GCCTCGAAAGAGTCCTGTATTG |  |  |
| *Pink1* | | Mouse | | Forward: TTCTTCCGCCAGTCGGTAG | 141 | |
|  |  |  |  | Reverse: CTGCTTCTCCTCGATCAGCC |  |  |
| *p53* | | Mouse | | Forward: GTGTGGTGCAGATCGCAGT | 100 | |
|  |  |  |  | Reverse: ATCATGCCTTCGGACTTGATG |  |  |
| *p16* | | Mouse | | Forward: ACATCAAGACATCGTGCGATATT | 78 | |
|  |  |  |  | Reverse: CCAGCGGTACACAAAGACCA |  |  |
| *p21* | | Mouse | | Forward: CGAGAACGGTGGAACTTTGAC | 107 | |
|  |  |  |  | Reverse: CCAGGGCTCAGGTAGACCTT |  |  |
| *Ctgf* | | Mouse | | Forward: CACTCTGCCAGTGGAGTTCA | 111 | |
|  |  |  |  | Reverse: AAGATGTCATTGTCCCCAGG |  |  |
| *Fibronectin* | | Mouse | | Forward: ACAAGGTTCGGGAAGAGGTT | 93 | |
|  |  |  |  | Reverse: CCGTGTAAGGGTCAAAGCAT |  |  |
| *a-Sma* | | Mouse | | Forward: GATGAAGCCCAGAGCAAGAG | 86 | |
|  |  |  |  | Reverse: CTTTTCCATGTCGTCCCAGT |  |  |
| *Tgf-β1* | | Mouse | | Forward: CCACCTGCAAGACCATCGAC | 91 | |
|  |  |  |  | Reverse: CTGGCGAGCCTTAGTTTGGAC |  |  |
| *Nf-kb* | | Mouse | | Forward: AGAGGGGATTTCGATTCCGC | 96 | |
|  |  |  |  | Reverse: CCTGTGGGTAGGATTTCTTGTTC |  |  |
| *Il-1β* | | Mouse | | Forward: GCTTCAGGCAGGCAGTATC | 133 | |
|  |  |  |  | Reverse: AGGATGGGCTCTTCTTCAAAG |  |  |
| *cGas* | | Mouse | | Forward: GAGGCGCGGAAAGTCGTAA | 98 | |
|  |  |  |  | Reverse: TTGTCCGGTTCCTTCCTGGA |  |  |
| *Sting* | | Mouse | | Forward: TATACCTCAGTTGGATGTTTGGC | 80 | |
|  |  |  |  | Reverse: CTGGAGTCAAGCTCTGAAGGC |  |  |
| *GAPDH* | | Human | | Forward: CGACCACTTTGTCAAGCTCA | 59 | |
|  |  |  |  | Reverse: CCCTGTTGCTGTAGCCAAAT |  |  |
| *PINK1* | | Human | | Forward: CCCAAGCAACTAGCCCCTC | 107 | |
|  |  |  |  | Reverse: GGCAGCACATCAGGGTAGTC |  |  |
| *p53* | | Human | | Forward: CAGCACATGACGGAGGTTGT | 125 | |
|  |  |  |  | Reverse: TCATCCAAATACTCCACACGC |  |  |
| *p16* | | Human | | Forward: GATCCAGGTGGGTAGAAGGTC | 74 | |
|  |  |  |  | Reverse: CCCCTGCAAACTTCGTCCT |  |  |
| *p21* | | Human | | Forward: TGTCCGTCAGAACCCATGC | 139 | |
|  |  |  |  | Reverse: AAAGTCGAAGTTCCATCGCTC |  |  |
| *CTGF* | | Human | | Forward: AAAAGTGCATCCGTACTCCCA | 109 | |
|  |  |  |  | Reverse: CCGTCGGTACATACTCCACAG |  |  |
| *FIBRONECTIN* | | Human | | Forward: GCGAGAGTGCCCCTACTACA | 70 | |
|  |  |  |  | Reverse: GTTGGTGAATCGCAGGTCA |  |  |
| *a-SMA* | | Human | | Forward: AAAAGACAGCTACGTGGGTGA | 75 | |
|  |  |  |  | Reverse: GCCATGTTCTATCGGGTACTTC |  |  |
| *TGF-β1* | | Human | | Forward: CAATTCCTGGCGATACCTCAG | 86 | |
|  |  |  |  | Reverse: GCACAACTCCGGTGACATCAA |  |  |
| *IL-1β* | | Human | | Forward: CCTTCCAGGATGAGGACATGA | 71 | |
|  |  |  |  | Reverse: TGAGTCACAGAGGATGGGCTC |  |  |
| *Acadm* | | Mouse | | Forward: AACACAACACTCGAAAGCGG | 75 | |
|  |  |  |  | Reverse: TTCTGCTGTTCCGTCAACTCA |  |  |
| *Slc25a12* | | Mouse | | Forward: CCAACTGTTTGACAAGAGCGG | 85 | |
|  |  |  |  | Reverse: GTGGTGATGGATAATAGTCTGCC |  |  |
| *Pdss2* | | Mouse | | Forward: GGCATAACCTACAACTGCGG | 102 | |
|  |  |  |  | Reverse: CACTGACCATGTCGTAGTTCTG |  |  |
| *Fbp1* | | Mouse | | Forward: CACCGCGATCAAAGCCATCT | 87 | |
|  |  |  |  | Reverse: CCAGTCACATTGGTTGAGCCA |  |  |
| *Acsf3* | | Mouse | | Forward: AGATTACCGTATTCATGGCAGTG | 75 | |
|  |  |  |  | Reverse: GCTGCGTGAAATGCTTGTCA |  |  |
| *Mthfr* | | Mouse | | Forward: AGATGAGGCGCAGAATGGAC | 107 | |
|  |  |  |  | Reverse: CATCCGGTCAAACCTGGAGAT |  |  |
| *Taldo1* | | Mouse | | Forward: GCCTCATCGAGCTTTACAAAGA | 89 | |
|  |  |  |  | Reverse: TCCAGCCTGAATCCCCTCC |  |  |
| *Pfkl* | | Mouse | | Forward: GGAGGCGAGAACATCAAGCC | 80 | |
|  |  |  |  | Reverse: GCACTGCCAATAATGGTGCC |  |  |
| *Acads* | | Mouse | | Forward: GACTGGCGACGGTTACACA | 87 | |
|  |  |  |  | Reverse: GGCAAAGTCACGGCATGTC |  |  |

**
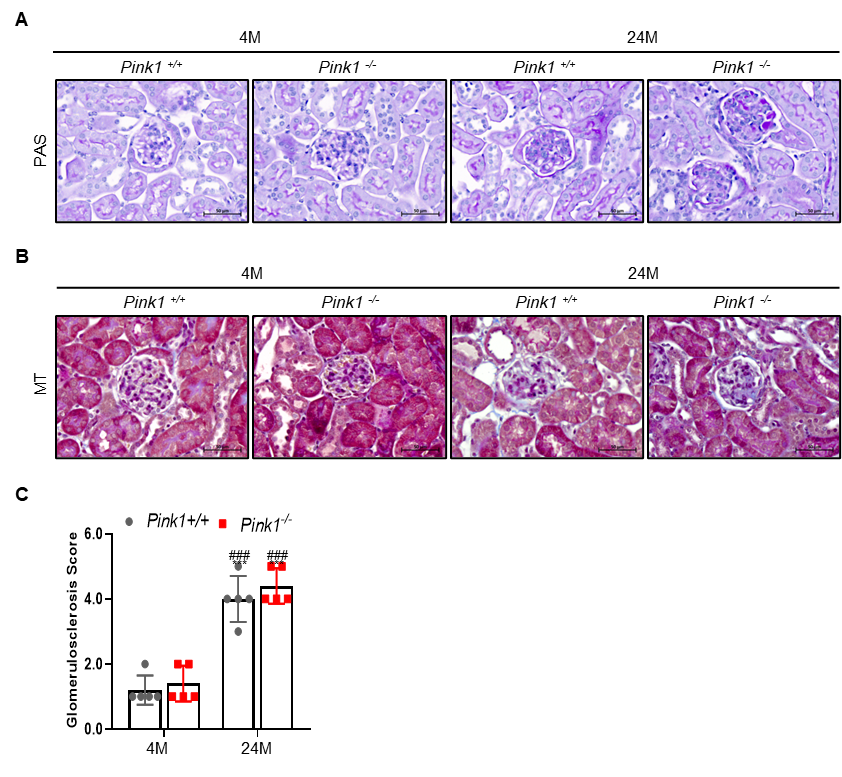
Supplemental Figure 1.** Representative of (A) periodic acid-Schiff-stain and (B) Masson’s Trichrome-stain for glomerular lesion of Pink1^+/+^ and Pink1^-/-^ mice kidneys at the age of 4 and 24 months. Scale bar = 50μm. (C) The quantification of glomerular lesions using glomerulosclerosis score. mean ± standard error of mean. *p < 0.05, **p < 0.01, ***p < 0.001 vs. Pink1^+/+^ 4M, # p < 0.05, ## p < 0.01, ### p < 0.001 vs Pink1^-/-^ 4M, M: months


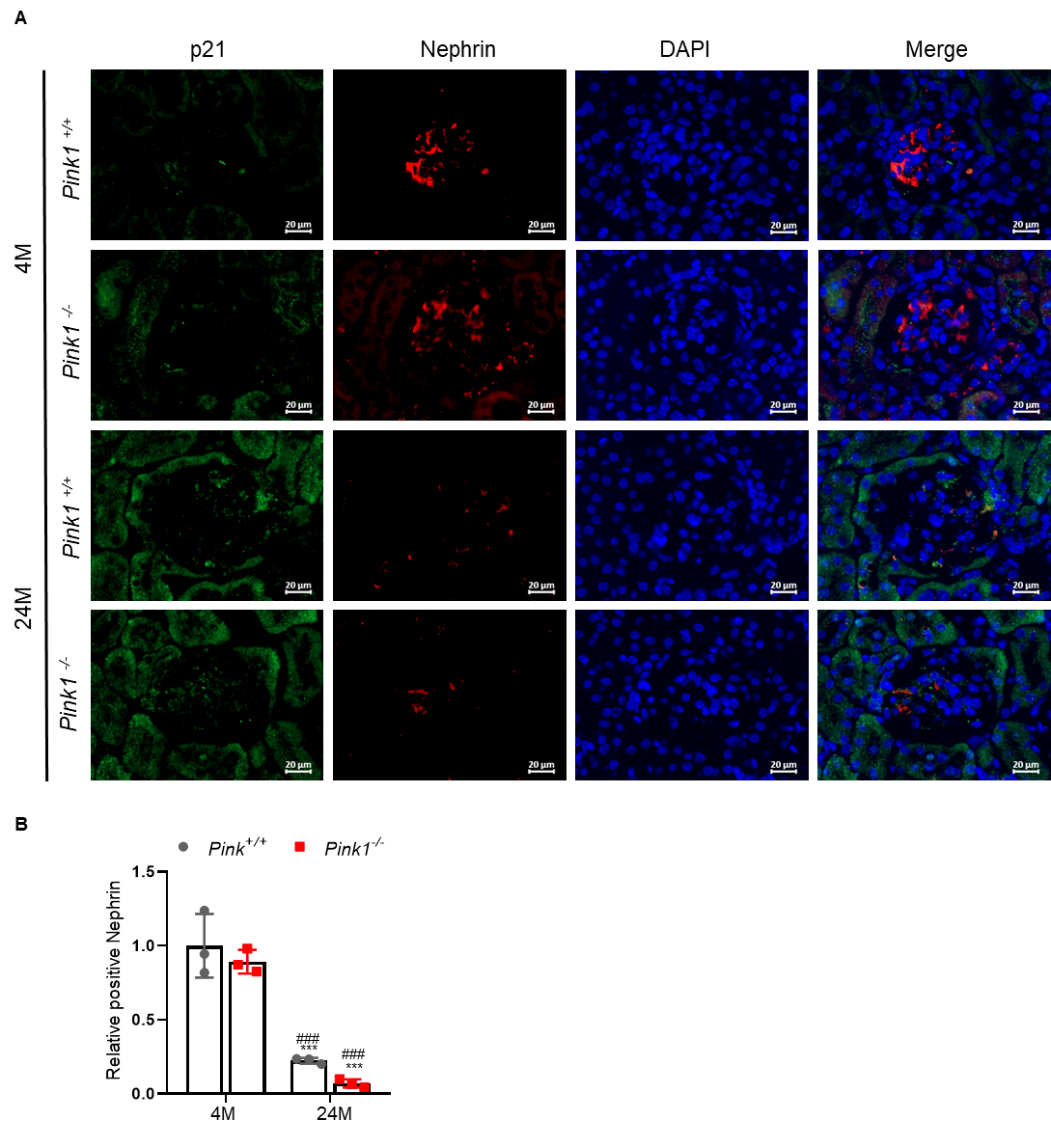


**Supplemental Figure 2.** (A) The immunofluorescence of Nephrin(green), p21(red) and DAPI stained nuclei (blue) of Pink1^+/+^ and Pink1^-/-^ mice kidneys at the age of 4 and 24 months. Scale bar = 20μm. (B) the quantification of Nephrin-positive podocyte cells. mean ± standard error of mean. *p < 0.05, **p < 0.01, ***p < 0.001 vs. Pink1^+/+^ 4M, # p < 0.05, ## p < 0.01, ### p < 0.001 vs. Pink1^-/-^ 4M, M: months


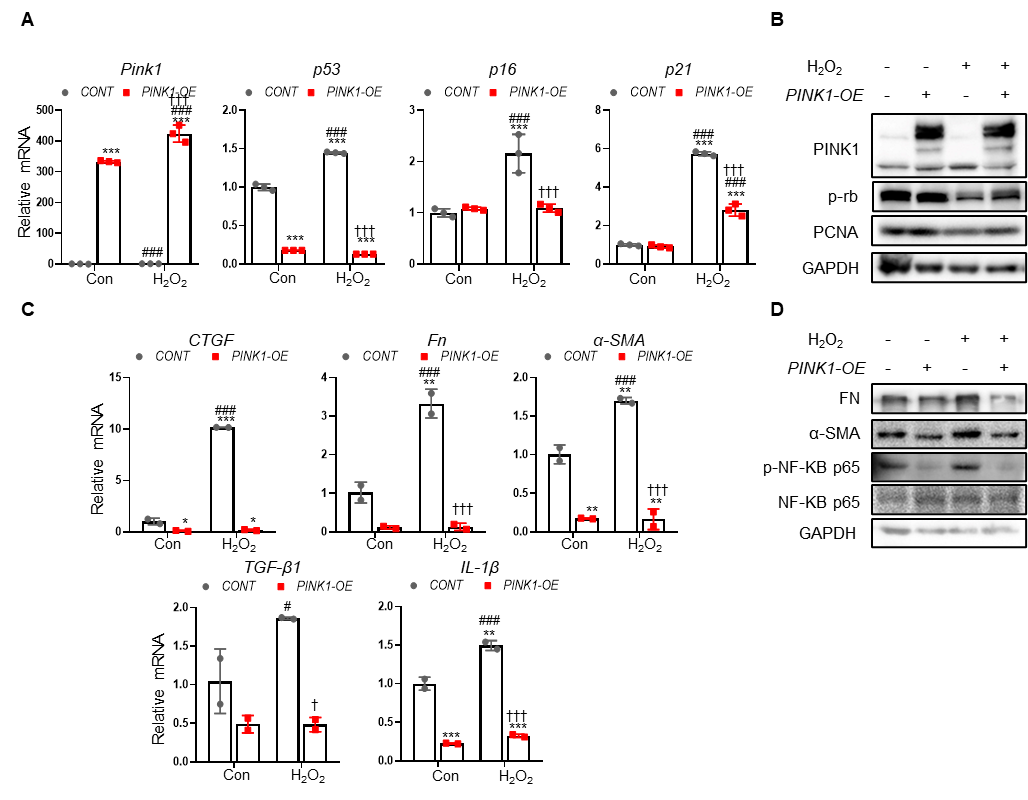


**Supplemental Figure 3.** (A) The mRNA levels of senescence signaling mediators, p53, p16, and p21, in CONT and PINK1-OE renal tubular epithelial cells (HKC-8) with H_2_O_2_ treatment. (B) Western blotting of cell cycle marker (p-Rb, PCNA) in CONT and PINK1-OE H_2_O_2_-treated HKC-8 cells. (C) The mRNA level of SASPs (CTGF, Fibronectin, α-SMA, TGF-β1, and IL-1β) in CONT and PINK1-OE H_2_O_2_-treated HKC-8 cells. (D) Western blotting of SASPs (Fibronectin, α-SMA, p-NF-kb-p65, and NF-kb p65) in CONT and PINK1-OE H_2_O_2_-treated HKC-8 cells. mean ± standard error of mean. (A, C) *p < 0.05, **p < 0.01, ***p < 0.001 vs. CONT without H_2_O_2_, ^#^ p < 0.05, ^##^ p < 0.01, ^###^ p < 0.001 vs. PINK1-OE without H_2_O_2_, **^†^** p < 0.05, **^††^** p < 0.01, **^†††^** p < 0.001 vs. CONT with H_2_O_2_, CONT: control, OE: overexpression


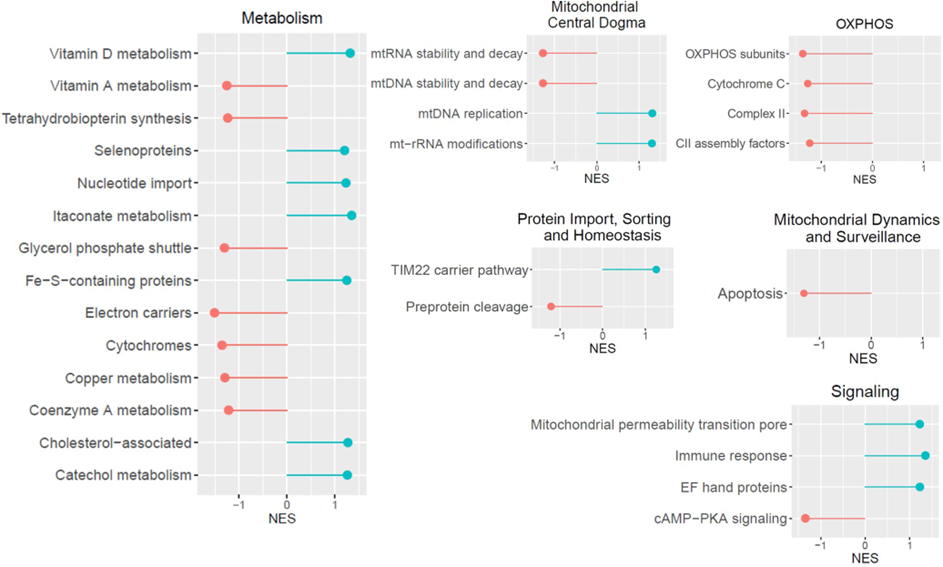
**Supplement Figure 4.** **Different mitochondrial localization between Pink1^-/-^ and Pink1^+/+^ 24-month-old mice.** Lollipop plots for statistically significant gene sets on mitochondrial localization, determined by fGSEA. The plots display different mitochondrial localization between Pink1^-/-^ and Pink1^+/+^ 24-month-old mice. The only pathways of FDR ≤ 0.25 are displayed. NES, nominal enrichment score.


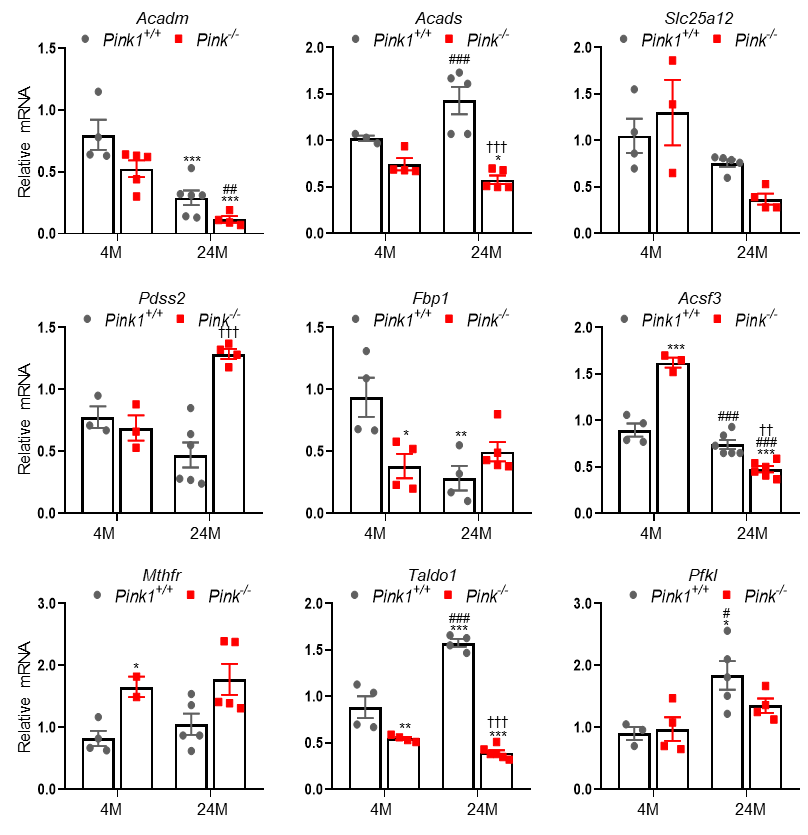


**supplemental Figure 5.** The mRNA level changes of Pink1^+/+^ and Pink1^-/-^ mice kidney tissue at 4 and 24 months. mean ± standard error of mean. *p < 0.05, **p < 0.01, ***p < 0.001 vs. Pink1^+/+^ 4M, ^#^ p < 0.05, ^##^ p < 0.01, ^###^ p < 0.001 vs. Pink1^-/-^ 4M, **^†^** p < 0.05, **^††^** p < 0.01, **^†††^** p < 0.001 vs. Pink1^+/+^ 24M, M: months

**7**
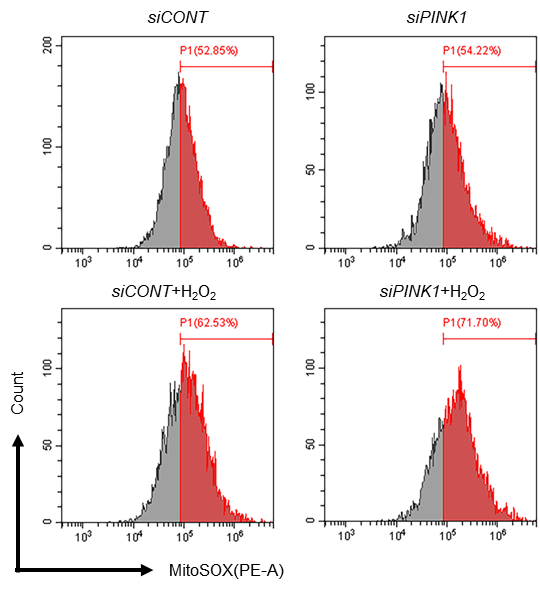


**Supplemental figure 6.** Measurement of mitochondrial ROS in *siPINK1* and H_2_O_2_ treated cells.


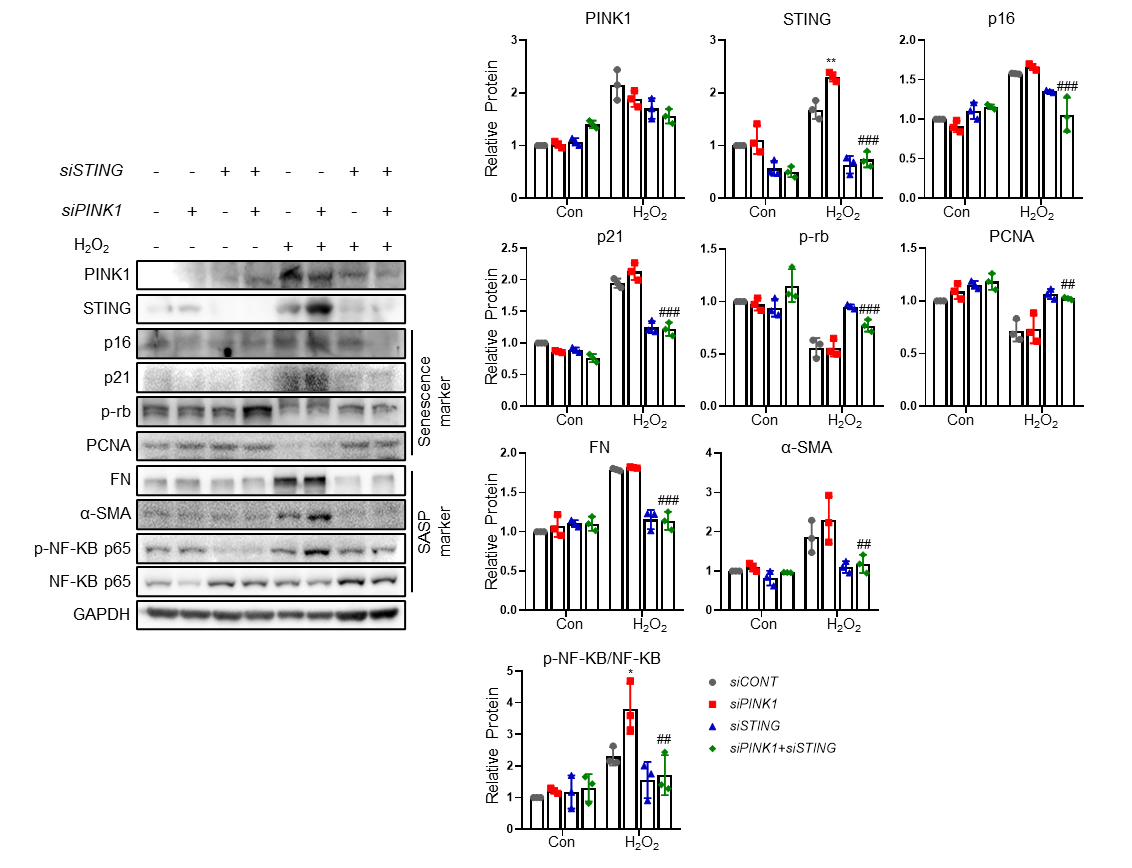


**supplemental Figure 7.** The change of senescence signaling mediators and SASPs in siPINK1, siSTING and H_2_O_2_ treated cells. mean ± standard error of mean. *p < 0.05, **p < 0.01, ***p < 0.001 vs. siCONT+H_2_O_2_, ^#^ p < 0.05, ^##^ p < 0.01, ^###^ p < 0.001 vs. siPINK1+ H_2_O_2_, CONT: control.


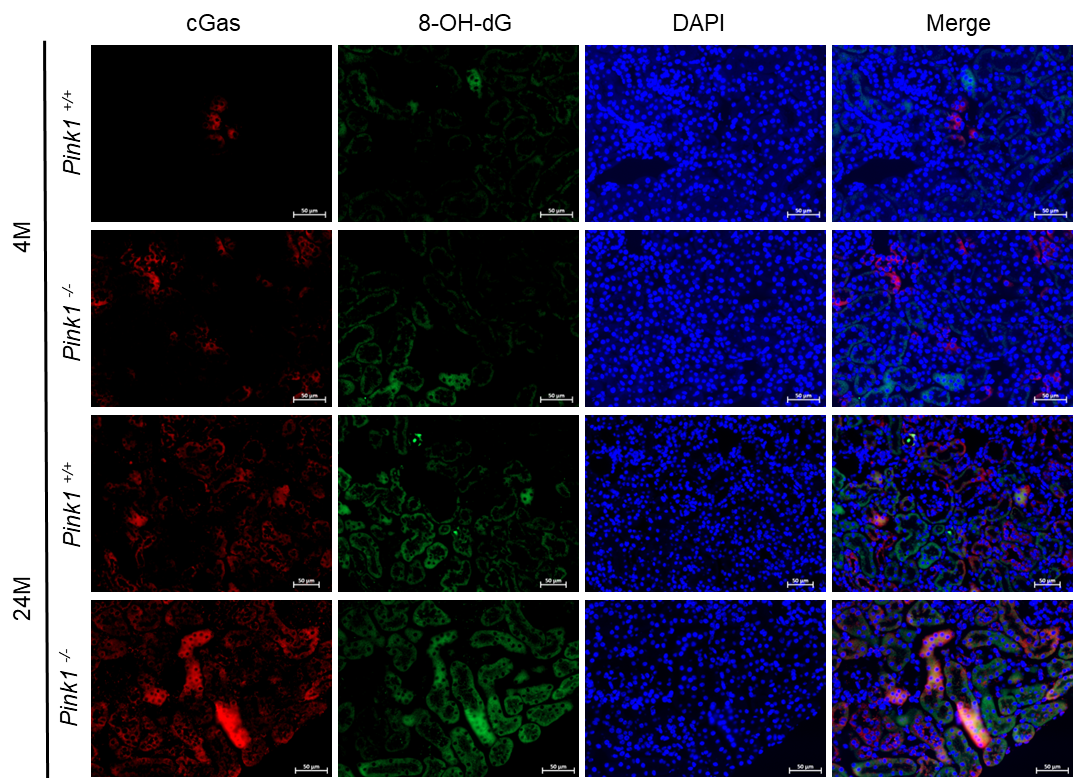


**supplemental Figure 8.** The immunofluorescence of 8-OH-dG(green) and cGAS(red) and DAPI stained nuclei (blue) of Pink1^+/+^ and Pink1^-/-^ mice kidneys at the age of 4 and 24 months. Scale bar = 50μm.
