# Supplementary figures and images for: PTEN‐induced kinase 1 is associated with renal aging, via the cGAS‐STING pathway
Source: Aging Cell. 2023 May 15;22(7):e13865. doi: 10.1111/acel.13865 (PMC10352563; doi:10.1111/acel.13865)

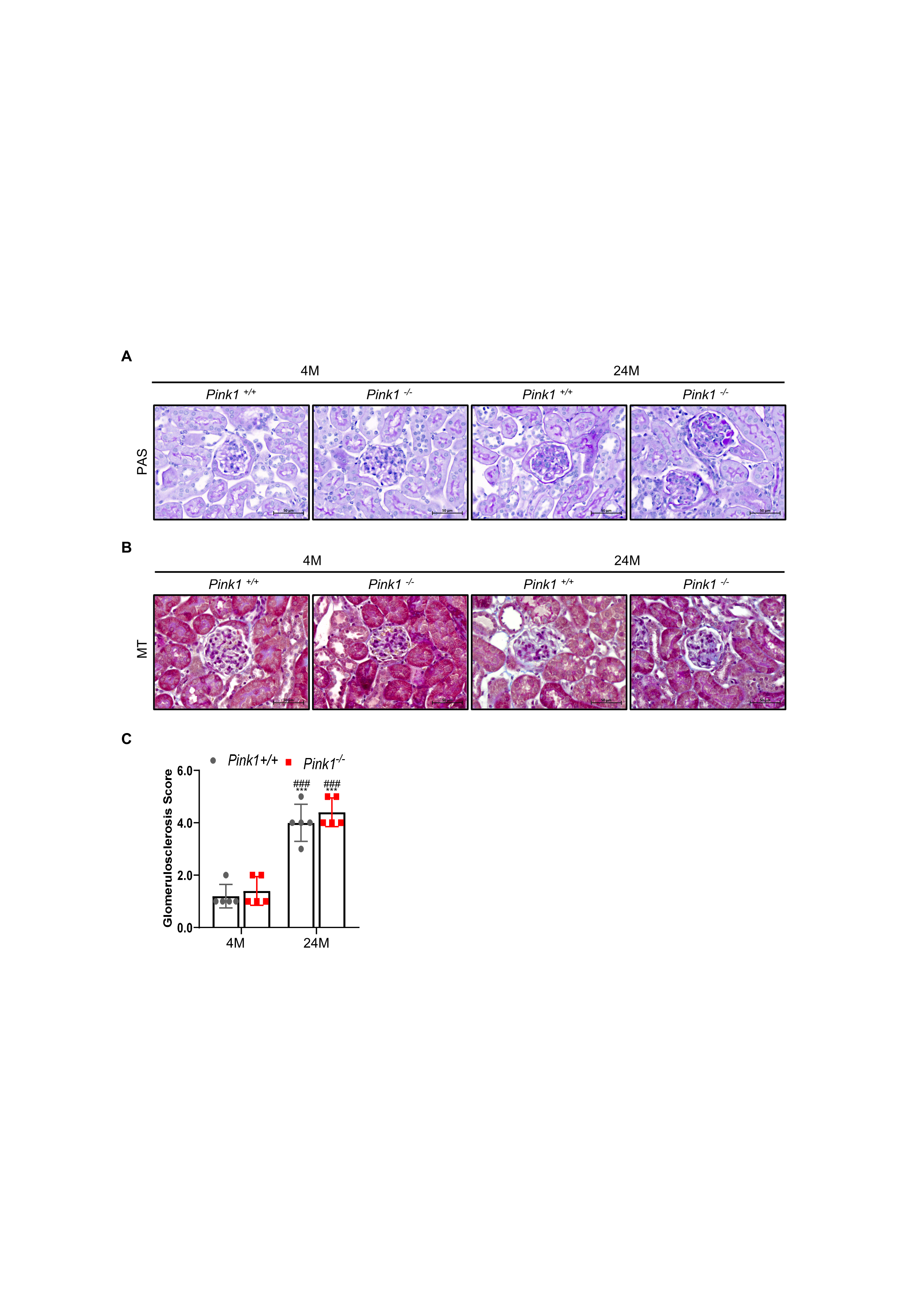

Supplement: Supplementary file 2 — Figure S1: [file ACEL-22-e13865-s002.tiff]

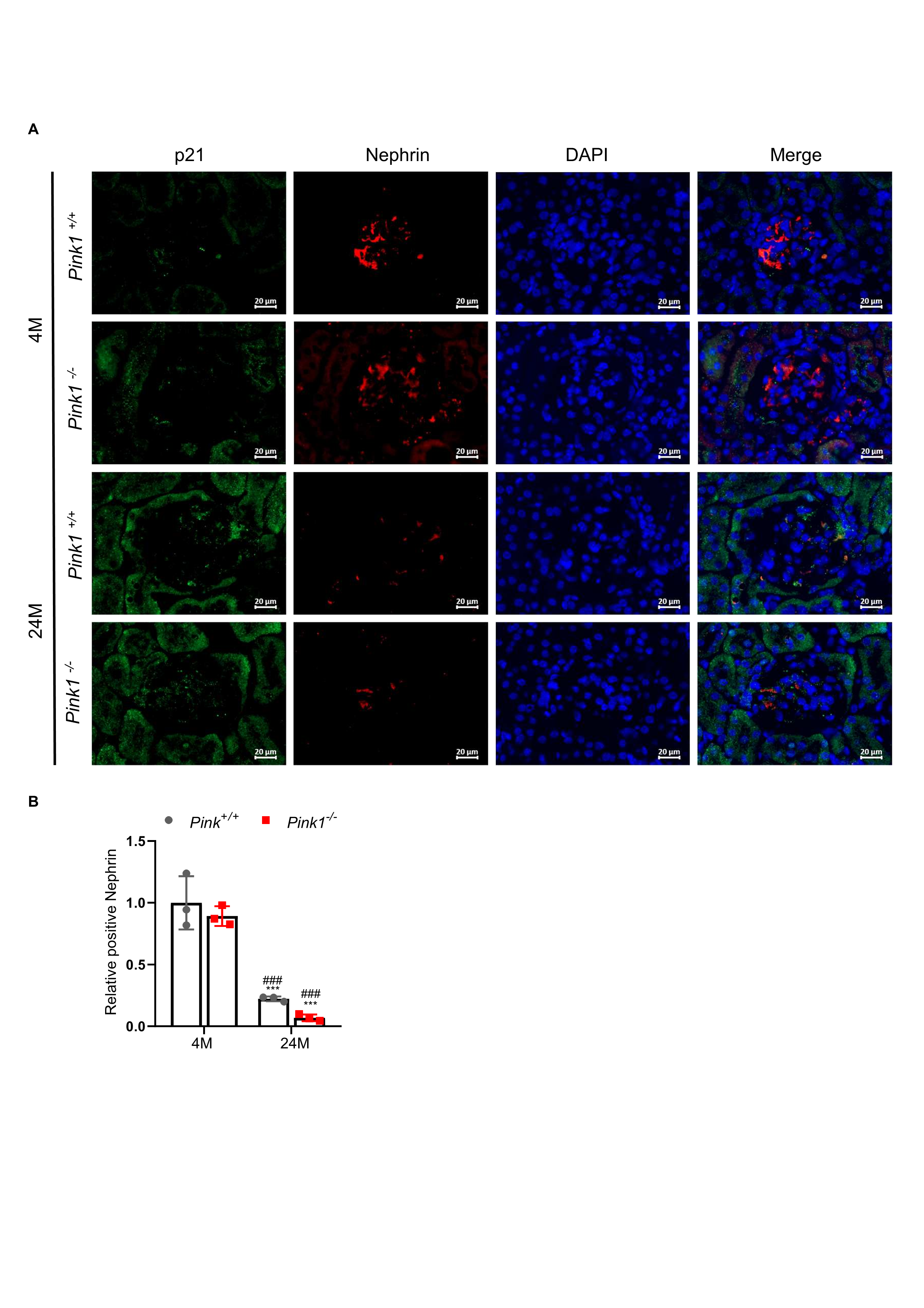

Supplement: Supplementary file 3 — Figure S2: [file ACEL-22-e13865-s007.tiff]

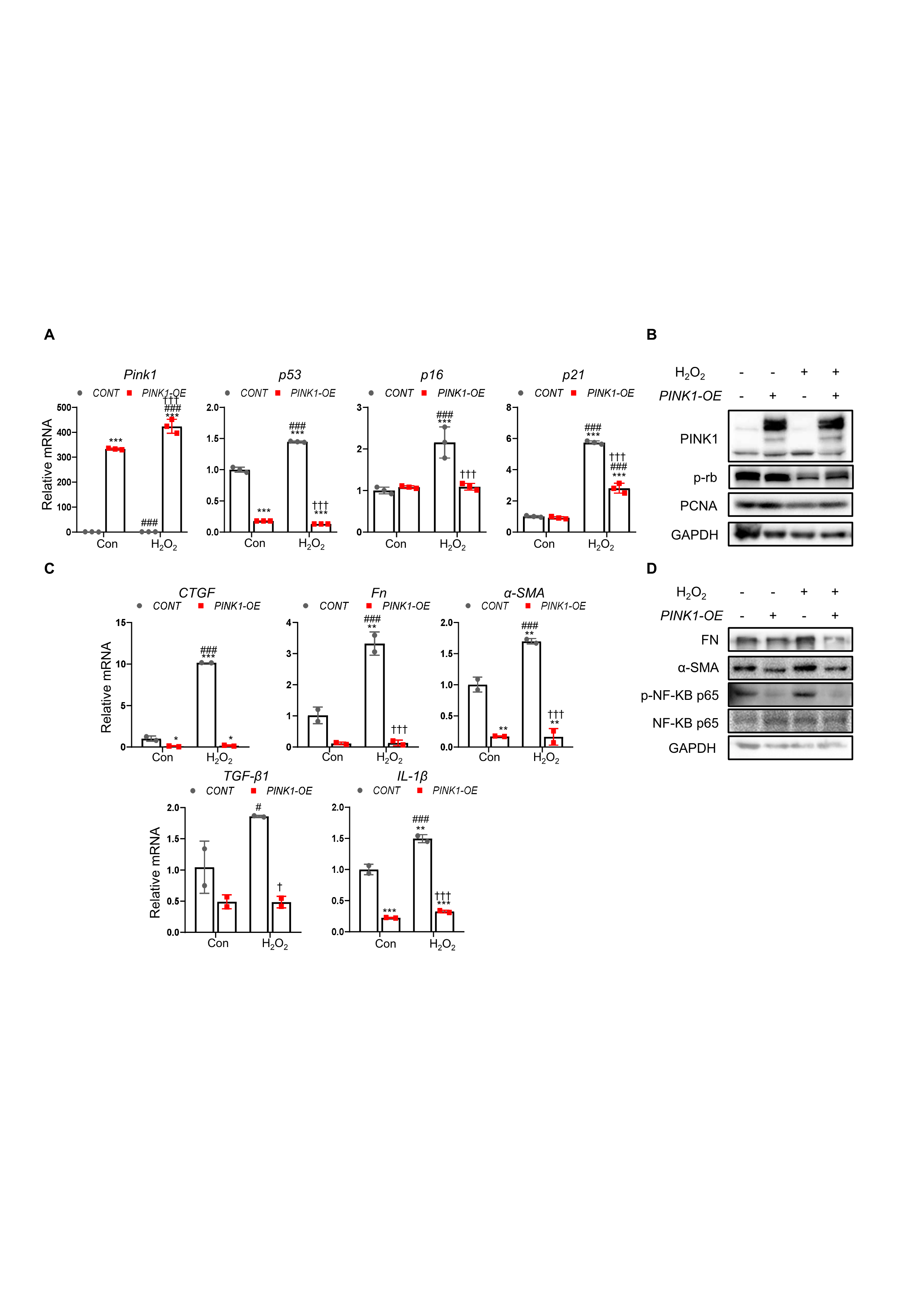

Supplement: Supplementary file 4 — Figure S3: [file ACEL-22-e13865-s009.tiff]

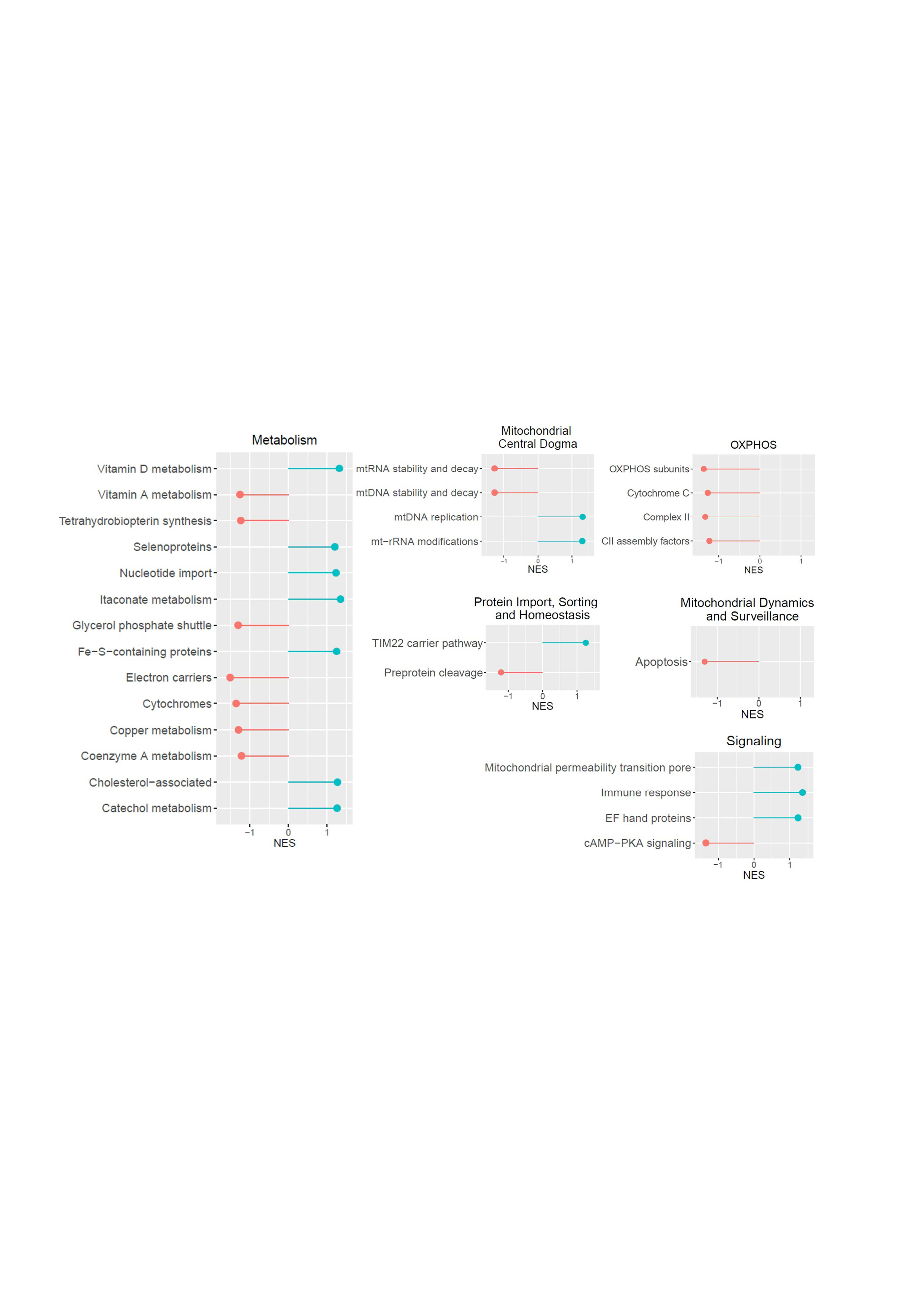

Supplement: Supplementary file 5 — Figure S4: [file ACEL-22-e13865-s004.tiff]

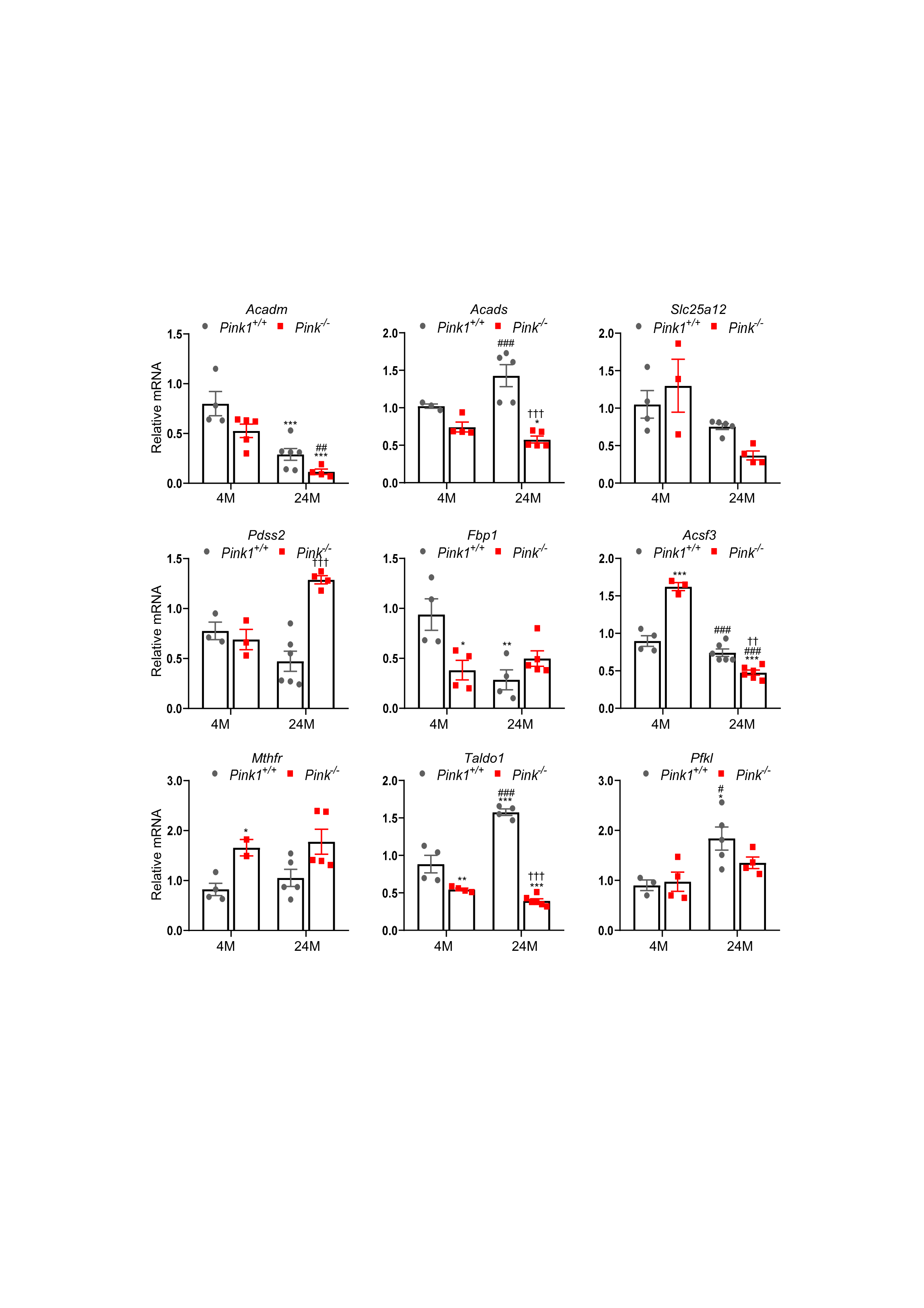

Supplement: Supplementary file 6 — Figure S5: [file ACEL-22-e13865-s001.tiff]

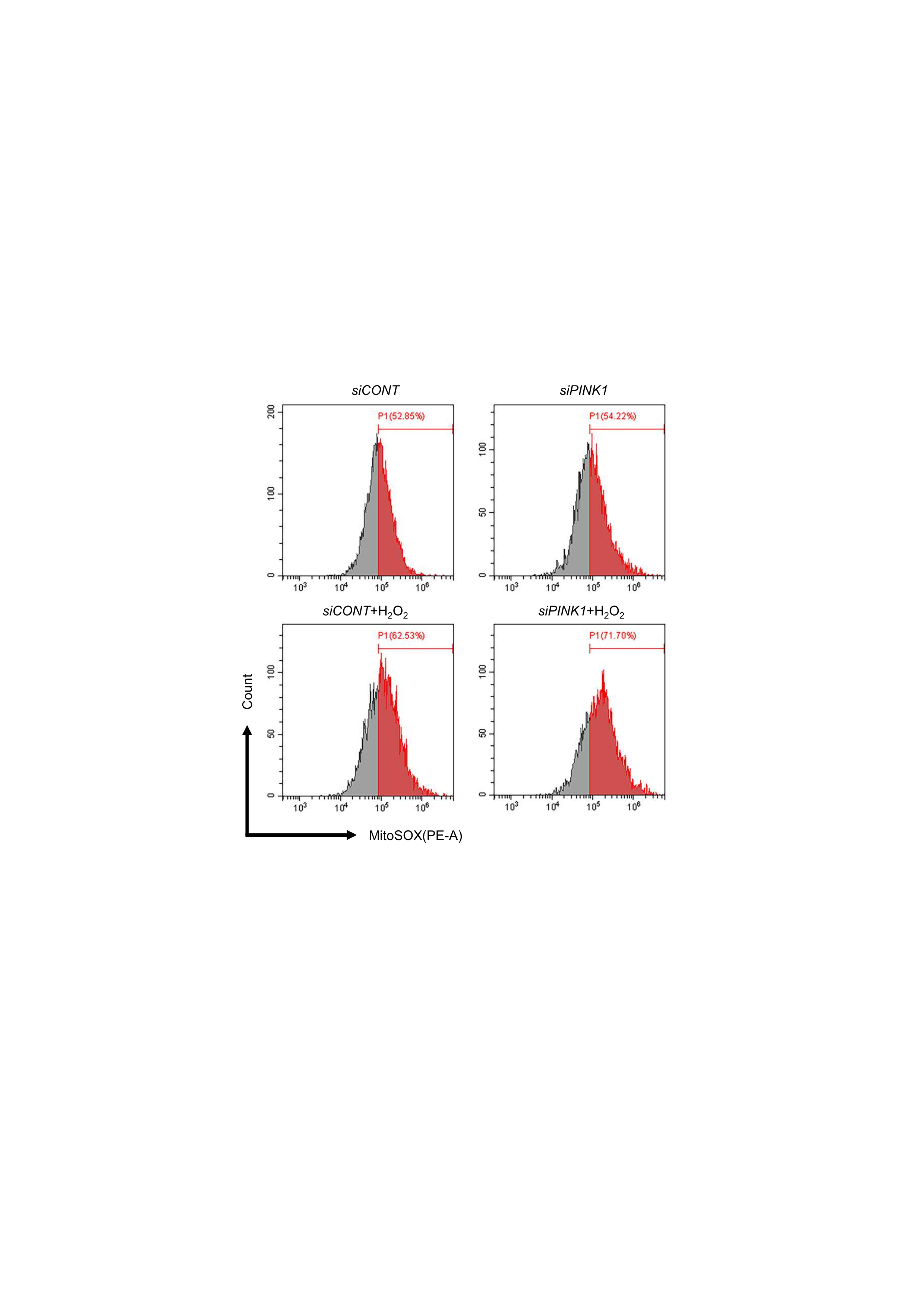

Supplement: Supplementary file 7 — Figure S6: [file ACEL-22-e13865-s008.tiff]

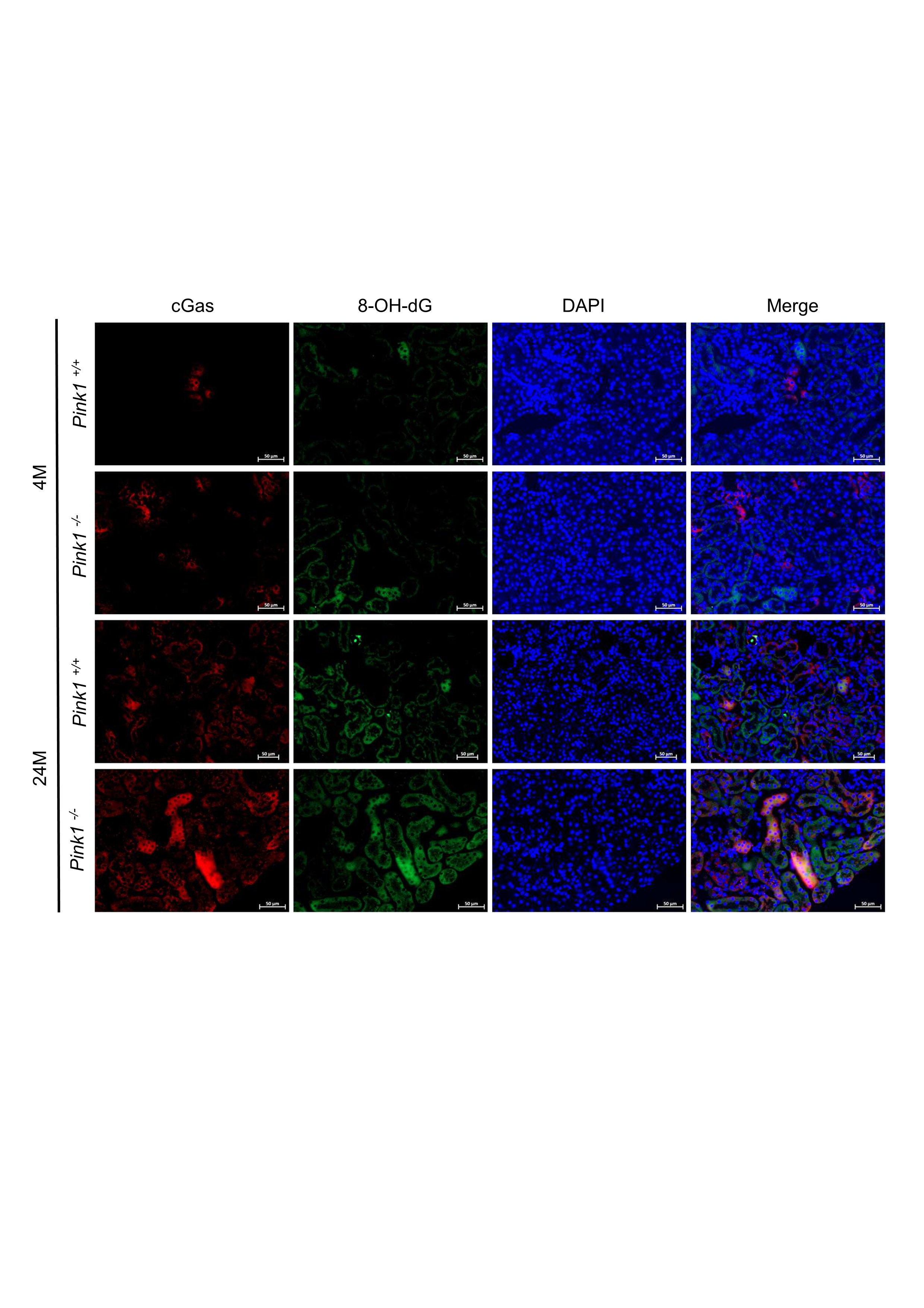

Supplement: Supplementary file 8 — Figure S7: [file ACEL-22-e13865-s005.tiff]

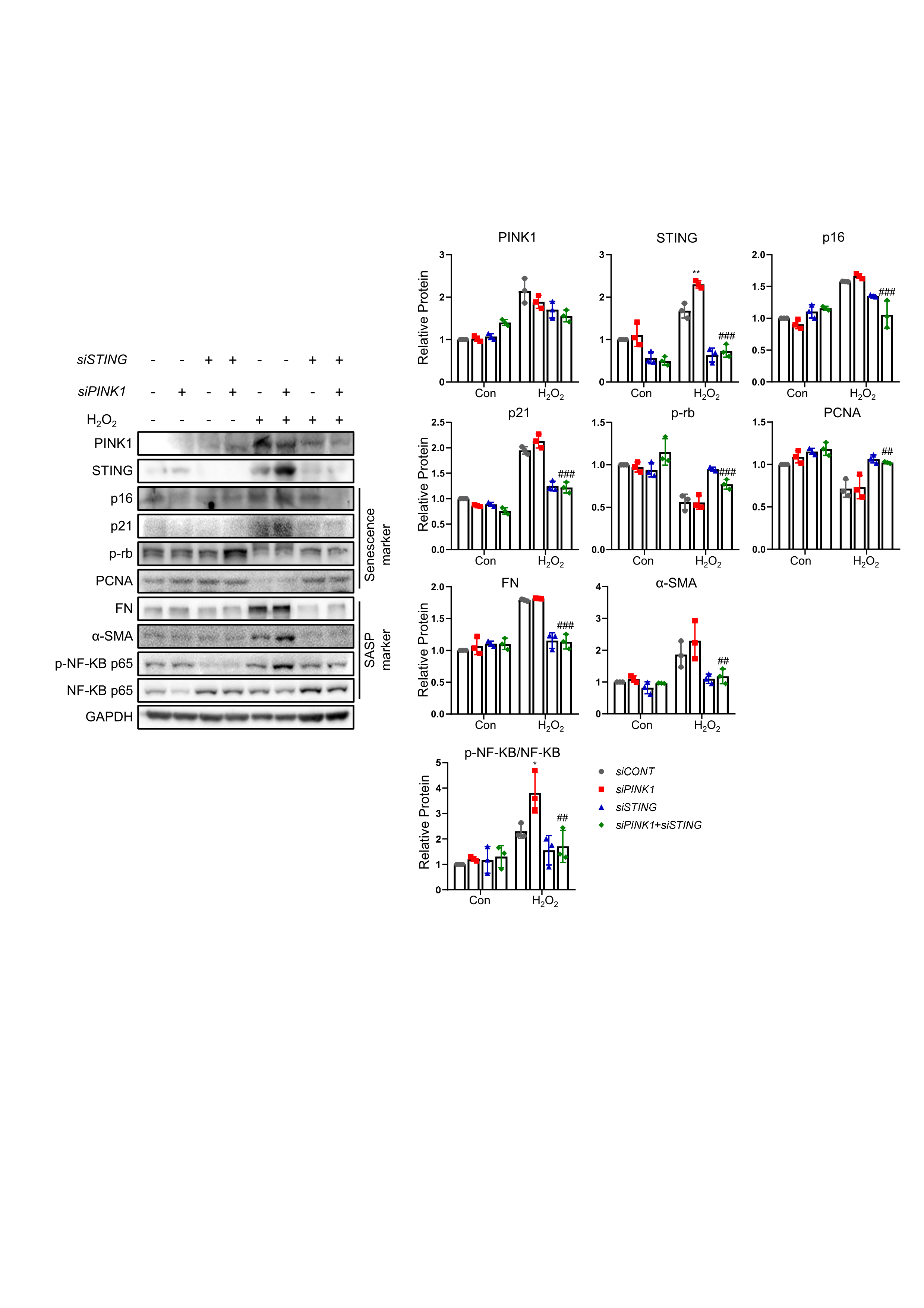

Supplement: Supplementary file 9 — Figure S8: [file ACEL-22-e13865-s006.tiff]
